# Osteopontin deficiency promotes cartilaginous endplate degeneration by enhancing the NF-κB signaling to recruit macrophages and activate the NLRP3 inflammasome

**DOI:** 10.1038/s41413-024-00355-3

**Published:** 2024-09-06

**Authors:** Yanqiu Wang, Wanqian Zhang, Yi Yang, Jinghao Qin, Ruoyu Wang, Shuai Wang, Wenjuan Fu, Qin Niu, Yanxia Wang, Changqing Li, Hongli Li, Yue Zhou, Minghan Liu

**Affiliations:** 1 Department of Orthopedics, Xinqiao Hospital, Army Medical University, Chongqing, China; 2 Experimental Center of Basic Medicine, College of Basic Medical Sciences, Army Medical University, Chongqing, China; 3 Institute of Pathology and Southwest Cancer Center, Southwest Hospital, Army Medical University, Chongqing, China

**Keywords:** Bone, Diseases

## Abstract

Intervertebral disc degeneration (IDD) is a major cause of discogenic pain, and is attributed to the dysfunction of nucleus pulposus, annulus fibrosus, and cartilaginous endplate (CEP). Osteopontin (OPN), a glycoprotein, is highly expressed in the CEP. However, little is known on how OPN regulates CEP homeostasis and degeneration, contributing to the pathogenesis of IDD. Here, we investigate the roles of OPN in CEP degeneration in a mouse IDD model induced by lumbar spine instability and its impact on the degeneration of endplate chondrocytes (EPCs) under pathological conditions. OPN is mainly expressed in the CEP and decreases with degeneration in mice and human patients with severe IDD. Conditional *Spp1* knockout in EPCs of adult mice enhances age-related CEP degeneration and accelerates CEP remodeling during IDD. Mechanistically, OPN deficiency increases CCL2 and CCL5 production in EPCs to recruit macrophages and enhances the activation of NLRP3 inflammasome and NF-κB signaling by facilitating assembly of IRAK1-TRAF6 complex, deteriorating CEP degeneration in a spatiotemporal pattern. More importantly, pharmacological inhibition of the NF-κB/NLRP3 axis attenuates CEP degeneration in OPN-deficient IDD mice. Overall, this study highlights the importance of OPN in maintaining CEP and disc homeostasis, and proposes a promising therapeutic strategy for IDD by targeting the NF-κB/NLRP3 axis.

## Introduction

Low back pain (LBP) is a very common symptom that affect people of all ages and is the leading cause of disability worldwide, causing enormous productivity loss and healthcare costs.^1,2^ While various abnormalities or diseases can cause LBP, intervertebral disc degeneration (IDD) has been recognized as a significant contributor.^3,4^ Pathologically, IDD is characterized by disc inflammation, extracellular matrix (ECM) disorder, loss of disc height, and ingrowth of nociceptive nerves and blood vessels.^5,6^ However, the pathological process of IDD has not been fully understood, and currently, clinical therapies for IDD are restricted to conservative medications and surgeries. In addition, these treatments merely alleviate clinical symptoms without restoring disc homeostasis.^7^ Therefore, understanding the molecular mechanisms underlying the pathogenic process of IDD will be of significance in developing novel biotherapeutics and medical strategies.

The intervertebral disc (IVD) is a diarthrodial joint which is composed of three unique tissues, including the central nucleus pulposus (NP) rich in proteoglycan, the peripheral annulus fibrosus (AF) with collagenous lamellae, and the superior and inferior cartilaginous endplates (CEP) adjacent to the vertebrae. This fibrocartilaginous structure provides a base for spinal stability and flexibility, permitting the absorption and transmission of mechanical loading.^8^ While the dysfunction of any tissue in the IVD can cause IDD, the majority of researches has concentrated on the physiology, pathology, and cellular heterogeneity of NP and AF.^9–12^ The findings have led to the development of therapeutic strategies based on stem cells,^13,14^ small molecules,^15^ extracellular vesicles,^16^ biomaterials,^17^ or gene therapy^18^ for IDD. It is well known that the CEP serves as a selective permeability barrier that allows the diffusion of nutrients, metabolites, water, and ions between avascular discs and vascularized vertebrae, and is crucial for maintaining IVD homeostasis.^19–21^ However, there are few studies on the CEP, and little is known on how the CEP dysfunction contributes to the pathogenesis of IDD. Hence, it is worthwhile to determine the precise mechanisms underlying the CEP degeneration during the process of IDD.

Osteopontin (OPN), encoded by the *Spp1* gene, is a highly expressed in the bone and other tissues and this phosphorylated glycoprotein is involved in many physiological and pathologic processes, such as inflammation, biomineralization, bone remodeling, cell survival, fibrosis, tumorigenesis, and metastasis.^22,23^ It can also regulate the development and progression of bone diseases, including osteoarthritis, osteoporosis, and osteosarcoma.^24–26^ Liu et al. proved that OPN effectively suppressed osteoarthritis development through an OPN/CD44/PI3K signaling.^26^ However, inhibiting OPN expression could significantly improve joint swelling, cartilage erosion and monocyte infiltration in rheumatoid arthritis via interrupting signaling pathways of Syk/PI3K/Akt and NF-κB.^24^ In view of the previous findings, most studies focus on the secreted form of OPN interacting with its receptors while neglect the roles of intracellular OPN during disease development, both of which could exert distinct effects in the signal transduction and cell fate. Hence, it is of great importance to decipher the functional pattern of OPN in IDD. Notably, a previous study has shown that the abnormal levels of OPN expression are strongly associated with the CEP degeneration.^27,28^ Recent single-cell RNA sequencing (scRNA-seq) results have suggested that OPN is a novel cue in the IVD microenvironment, and it is related to the development and degeneration of IVD.^29^ Another scRNA-seq analysis of cells from degenerating and non-degenerating IVDs has proposed that the *Spp1* is a predictor of IDD.^30^ Furthermore, OPN has been suggested to be a pathogenic factor influencing the CEP homeostasis by modulating bone formation or angiogenesis, and a novel therapeutic target for degenerative CEP.^31^ Nevertheless, the specific mechanisms by which OPN regulates the CEP homeostasis remain obscure and have not been determined in vivo.

In this study, the impacts of conditional *Spp1* knockout on the CEP structure and function were tested in OPN deficient mice. The results revealed that OPN deficiency enhanced the NF-κB signaling activation in endplate chondrocytes (EPCs) and increased CCL2 and CCL5 production to recruit macrophages and activate the NLRP3 inflammasome, leading to CEP degeneration. Therapeutically, pharmacological inhibition of the NF-κB/NLRP3 axis attenuated the CEP degeneration and IDD development in conditional OPN-deficient mice. Therefore, our findings disclosed a cellular and molecular mechanism by which OPN functioned to maintain the CEP homeostasis and suggest a feasible therapeutic strategy for IDD.

## Results

### OPN expression is dramatically decreased in the CEP during degeneration

**Fig. 1:**
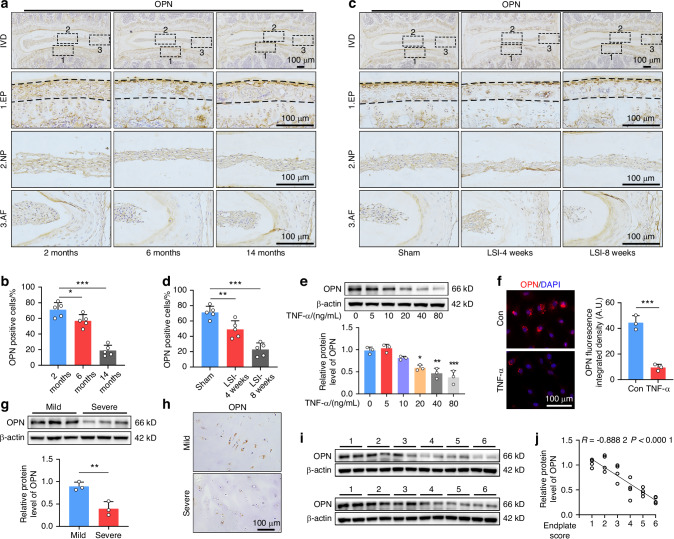
OPN expression is dramatically decreased in the CEP during degeneration. **a**, **b** IHC staining and quantification of OPN expression in the IVD tissues from 2, 6, and 14-month-old mice (*n* = 5). **c**, **d** IHC staining and quantification of OPN expression in the IVD tissues from the mice at 4- and 8-weeks post LSI surgery or the sham group (*n* = 5). **e** WB analysis of OPN expression in EPCs that had been treated with the indicated doses of TNF-α (*n* = 3). **f** IF staining and quantification of OPN expression in EPCs treated with, or without, TNF-α (80 ng/mL) (*n* = 3). **g** WB analysis of OPN expression in CEP tissues from patients with mild or severe degeneration (*n* = 3). **h** IHC staining of OPN expression in CEP tissues from patients with mild or severe degeneration. **i**, **j** WB analysis of OPN expression in human CEP tissues and their correlation with the severity of degeneration (*n* = 24). *P < 0.05, **P < 0.01, ***P < 0.001

To explore the possible role of OPN in the pathogenesis of IDD, OPN expression was characterized in IVD tissues from mice and patients. Immunohistochemistry (IHC) analysis of mouse lumbar discs displayed that the expression level of OPN significantly decreased with aging, particularly in the CEP (Fig. 1a, b). Furthermore, following induction of lumbar spine instability (LSI), a commonly used model in CEP degeneration,^32^ the quantity of OPN-positive cells in the CEP decreased dramatically at 4- and 8-weeks post LSI surgery compared to the sham surgery in a time-dependent trend (Fig. 1c, d). Given that proinflammatory cytokines are crucial for the progression of IDD,^3^ next, we tested whether treatment with different doses of TNF-α could modulate OPN expression in EPCs. Western blot (WB) and immunofluorescence (IF) analyses exhibited that TNF-α treatment significantly reduced the relative levels of OPN expression in a dose-dependent way in EPCs (Fig. 1e, f). Clinically, CEP tissues were collected from IDD patients and divided into the mild and severe groups, based on the severity of endplate (EP) damage.^33^ Similarly, the levels of OPN expression in the CEP from the severe group of IDD patients were markedly lower than that in the mild group (Fig. 1g, h). Moreover, the relative levels of OPN in the CEP were negatively correlated with EP scores in this population of IDD patients (Fig. 1i, j). Hence, OPN is mainly expressed in the CEP of IVD and decreases during degeneration, suggesting a potentially pivotal role of OPN in the CEP homeostasis and IDD.

### OPN deficiency deteriorates the age-related CEP degeneration

**Fig. 2:**
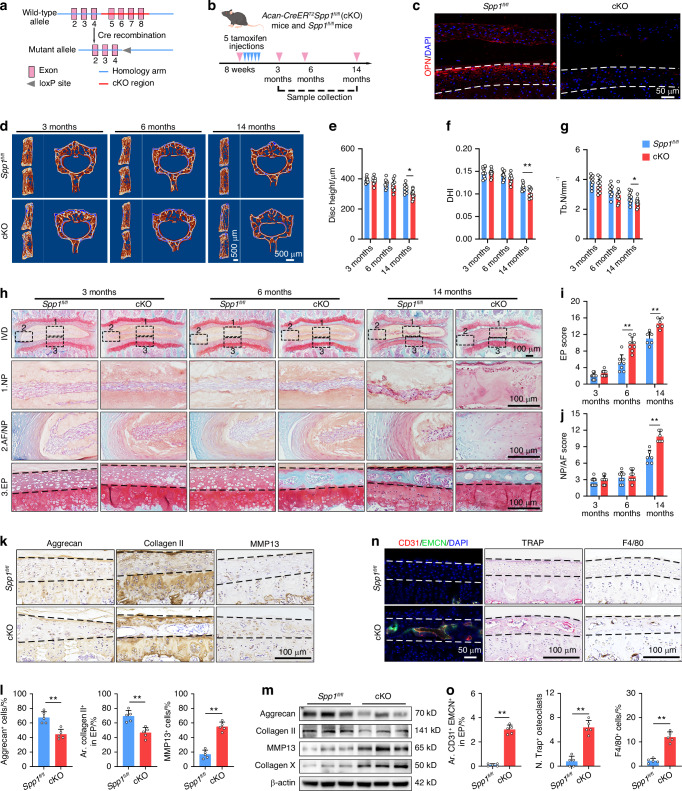
OPN deficiency deteriorates the age-related CEP degeneration. **a** A diagram illustrates the construction strategy for generating *Spp1* cKO mice. **b** A schematic graph of the experimental design. **c** IF staining of OPN expression in the IVD tissues from 3-month-old *Spp1*^*fl/fl*^ and cKO mice. **d** μCT reconstruction images of the hemi-section of a lumbar motion segment and the cross-section of a lumbar vertebral body from 3, 6, and 14-month-old *Spp1*^*fl/fl*^ and cKO mice. Quantification of μCT analyses for the disc height (**e**), disc height index (DHI; **f**), and trabecular number (Tb. N; **g**) of the mouse lumbar vertebrae from 3, 6, and 14-month-old *Spp1*^*fl/fl*^ and cKO mice (*n* = 10). SO-FG staining (**h**) and histological scores of lumbar EP (**i**) and NP/AF (**j**) in 3 (*n* = 10), 6 (*n* = 8), and 14-month-old (*n* = 6) *Spp1*^*fl/fl*^ and cKO mice. **k**, **l** IHC staining and quantification of Aggrecan, Collagen II, and MMP13 expression in CEP tissues from 6-month-old *Spp1*^*fl/fl*^ and cKO mice (*n* = 5). **m** WB analysis of Aggrecan, Collagen II, MMP13, and Collagen X expression in CEP tissues from 6-month-old *Spp1*^*fl/fl*^ and cKO mice (*n* = 3). **n** IF staining of CD31^+^ (red) EMCN^+^ (green) blood vessels (left), TRAP (magenta) staining (middle), and IHC staining of F4/80^+^ cells (right) in CEP tissues from 6-month-old *Spp1*^*fl/fl*^ and cKO mice. **o** Quantification of CD31^+^EMCN^+^ blood vessels (left), TRAP^+^ cells (middle), and F4/80^+^ cells (right) in CEP tissues from 6-month-old *Spp1*^*fl/fl*^ and cKO mice (*n* = 5). *P < 0.05, **P < 0.01

To better understand the role of OPN in vivo, conditional *Spp1* knockout mice were generated and used in the following experiments (Fig. S1A). *Acan-CreER*^*T2*^*Spp1*^*fl/fl*^ male mice at 8 weeks of age were intraperitoneally administered with tamoxifen for 5 days (referred to as “cKO” herein), which were then compared with age-matched *Spp1*^*fl/fl*^ mice (Fig. 2a, b). IF staining showed strong expression of OPN in the CEP of *Spp1*^*fl/fl*^ mice, which was dramatically reduced in that of the cKO group (Fig. 2c). The WB results further verified the Cre-mediated deletion of OPN in the CEP of cKO mice (Fig. S1J). Micro-computed tomography (μCT) analysis exhibited no obvious alteration in trabecular and cortical bone architecture between the *Spp1*^*fl/fl*^ and cKO mice at 3 and 6 months of age (Fig. 2d and Fig. S1B–D). However, the disc height and disc height index (DHI) became significantly less in the cKO mice than that in the *Spp1*^*fl/fl*^ mice at 14 months old (Fig. 2e, f). Likewise, the trabecular number of lumbar vertebrae in 14-month-old mice significantly decreased in the cKO versus the *Spp1*^*fl/fl*^ specimens (Fig. 2g). Next, Safranine O-Fast Green staining displayed no significant change in the IVD structural integrity of 3-month-old cKO mice, relative to the age-matched *Spp1*^*fl/fl*^ mice (Fig. 2h). Notably, the CEP began to ossify at 6 months of age and was largely replaced by bone tissue in 14-month-old cKO mice (Fig. 2h). Besides, the degrees of CEP ossification in the cKO mice were age-dependent (Fig. S1E). However, compared with the *Spp1*^*fl/fl*^ mice, the 14-month-old cKO mice exhibited more severe degenerative changes in the NP/AF, including cell loss, disorganized AF lamellae, signs of fibrosis, and boundary disruption of NP/AF (Fig. 2h). Accordingly, EP histological scores were significantly higher in the cKO mice than those in the *Spp1*^*fl/fl*^ mice at 6 and 14 months of age, while the NP/AF histological scores were higher in the cKO mice than those in the *Spp1*^*fl/fl*^ mice only at 14 months of age (Fig. 2i, j), indicating that CEP degeneration preceded the degeneration of NP/AF in OPN-deficient IVD. Therefore, subsequent experiments focused on the role of OPN in CEP homeostasis.

IHC staining displayed that OPN deficiency decreased the levels of Aggrecan and type II collagen (Collagen II) expression in the CEP at 6 months of age, suggesting an impaired anabolism of the ECM (Fig. 2k, l). In contrast, OPN deficiency increased the levels of matrix metalloproteinase 13 (MMP 13) and type X collagen (Collagen X) expression, compared with the *Spp1*^*fl/fl*^ mice, implying the occurrence of chondrocyte hypertrophy and ECM catabolism in the CEP of cKO mice (Fig. 2k, l and Fig. S1F). A similar pattern of Aggrecan, Collagen II, MMP13, and Collagen X expression was detected in the CEP of different groups of mice by WB (Fig. 2m and Fig. S1G). In addition, there was a trend of time-dependent decrease in the number of EPCs in the CEP and the number of EPCs in the CEP of cKO mice was significantly less than that in the age-matched *Spp1*^*fl/fl*^ mice at 6 and 14 months of age, while the number of TUNEL^+^ EPCs in the CEP of cKO mice were significantly greater than that in the *Spp1*^*fl/fl*^ mice at 6 months of age (Fig. S1H, I). Given that vascular ingrowth, high osteoclast activity, and macrophage infiltration can disturb the CEP homeostasis,^34,35^ we investigated whether any of these factors contributed to the CEP degeneration in OPN-deficient IVD. Intriguingly, type H blood vessels (CD31^+^EMCN^+^), which have been recognized as a contributor to endplate sclerosis,^34^ sprouted into the porous endplates of 6-month-old cKO mice, but not into that of *Spp1*^*fl/fl*^ mice (Fig. 2n, o). Furthermore, the quantity of tartrate-resistant acid phosphatase (TRAP) positive osteoclasts and F4/80^+^ macrophages in the endplates significantly increased in the cKO mice, relative to the *Spp1*^*fl/fl*^ mice (Fig. 2n, o). Therefore, OPN deficiency deteriorated the age-related degeneration in the CEP, including ECM breakdown, cell death, neovascularization, osteoclast activity, and macrophage invasion, directly impairing CEP homeostasis during IDD development.

### OPN deficiency accelerates CEP remodeling in mice following LSI

**Fig. 3:**
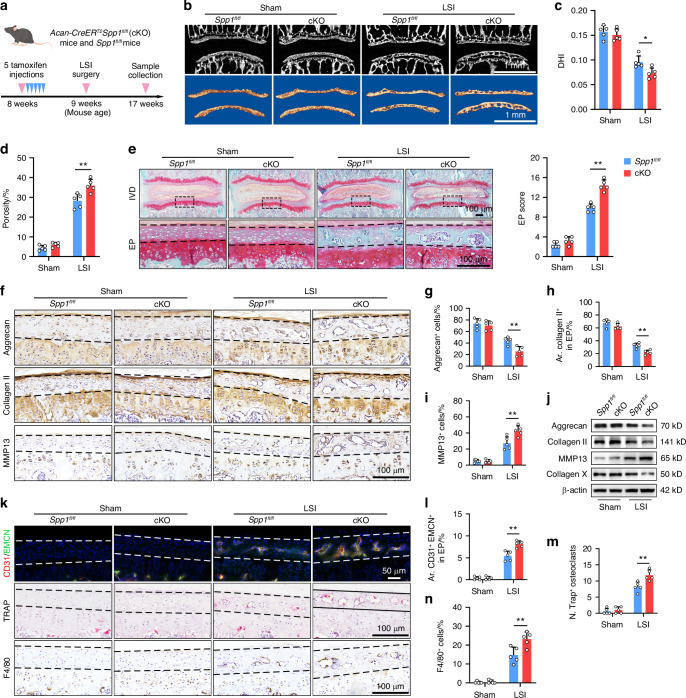
OPN deficiency accelerates CEP remodeling in mice following LSI. **a** A schematic graph of the experimental design. **b** μCT (top) and reconstruction (bottom) images of the coronal plane of lumbar endplates in *Spp1*^*fl/fl*^ and cKO mice with a sham or LSI surgery. Quantification of μCT analyses for DHI (**c**) and the porosity (**d**) of mouse lumbar endplates in *Spp1*^*fl/fl*^ and cKO mice with sham or LSI surgery (*n* = 5). **e** SO-FG staining and histological scores of lumbar EP in *Spp1*^*fl/fl*^ and cKO mice with a sham or LSI surgery (*n* = 5). **f**–**i** IHC staining and quantification of Aggrecan, Collagen II, and MMP13 expression in CEP tissues from *Spp1*^*fl/fl*^ and cKO mice with a sham or LSI surgery (*n* = 5). **j** WB analysis of Aggrecan, Collagen II, MMP13, and Collagen X expression in CEP tissues from *Spp1*^*fl/fl*^ and cKO mice with a sham or LSI surgery. **k** IF staining of CD31^+^ (red) EMCN^+^ (green) blood vessels (top), TRAP (magenta) staining (middle), and IHC staining of F4/80 (bottom) in CEP tissues from *Spp1*^*fl/fl*^ and cKO mice with a sham or LSI surgery. Quantification of CD31^+^EMCN^+^ blood vessels (**l**), TRAP^+^ cells (**m**), and F4/80^+^ cells (**n**) in CEP tissues from *Spp1*^*fl/fl*^ and cKO mice with a sham or LSI surgery (*n* = 5). *P < 0.05, **P < 0.01

Subsequently, we evaluated the impact of OPN deficiency on CEP degeneration in the LSI model (Fig. 3a and Fig. S2E). μCT analysis displayed that the space between vertebral bodies decreased at 8 weeks post LSI (Fig. 3b). Compared with the sham surgery, the DHI in the LSI-treated mice was significantly lower than that in the mice with sham surgery and following LSI, the DHI in the cKO mice was significantly lower than that in the *Spp1*^*fl/fl*^ mice (Fig. 3c). Moreover, analysis of three-dimensional reconstruction images of endplates revealed that percentages of porosity in the LSI-treated cKO mice were significantly higher than that in the LSI-treated *Spp1*^*fl/fl*^ mice (Fig. 3b, d). Consistently, Safranine O-Fast Green staining unveiled that the endplates from the LSI-treated cKO mice displayed more cavities surrounded by the green-stained bone matrix with higher histological scores, compared with that in the LSI-treated *Spp1*^*fl/fl*^ mice (Fig. 3e). Similarly, IHC staining and WB indicated that LSI surgery decreased the levels of Aggrecan and Collagen II, but increased MMP13 expression, relative to those in the mice with sham surgery, promoting ECM degradation, which were aggravated in the LSI-treated cKO mice (Fig. 3f–j and Fig. S2B). However, the levels of Collagen X expression markedly decreased in the LSI-treated mice, indicating fewer hypertrophic chondrocytes in ossified endplates, particularly in the LSI-treated cKO mice (Fig. 3j and Fig. S2A, B). Besides, the number of EPCs significantly decreased in LSI mice, accompanied by the increased number of apoptotic cells (Fig. S2C, D). Importantly, LSI mice exhibited abnormal angiogenesis with a progressive invasion of type H blood vessels into the porous endplates and an increase in the number of osteoclasts and macrophages, which were also exacerbated in the LSI-treated cKO mice (Fig. 3k–n). Thus, these findings suggest that OPN deficiency accelerates CEP remodeling in LSI mice.

### OPN-deficient EPCs enhances CCL2 and CCL5 expression to recruit macrophages

**Fig. 4:**
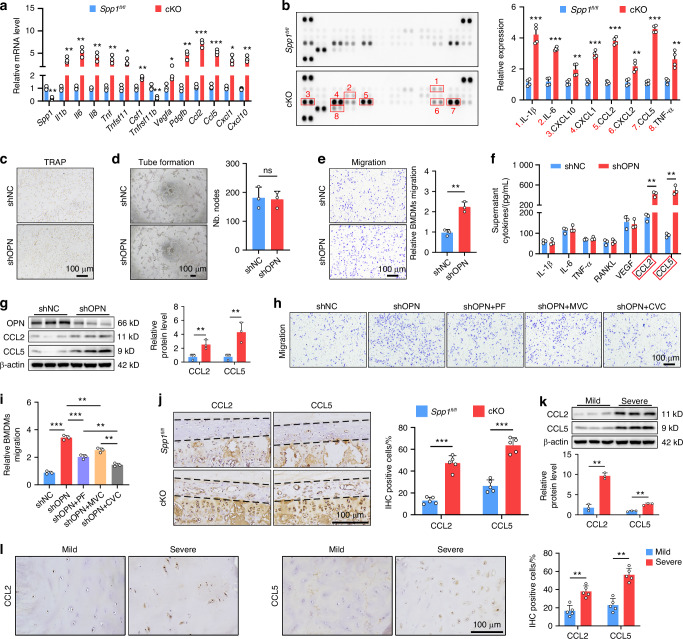
OPN-deficient EPCs enhances CCL2 and CCL5 expression to recruit macrophages. **a** RT-qPCR analysis of the relative levels of *Spp1*, *Il1b, Il6, Il8, Tnf*, *Tnfsf11, Csf1, Tnfsf11b*, *Vegfa, Pdgfb*, *Ccl2, Ccl5, Cxcl1*, and *Cxcl10* mRNA transcripts in CEP tissues from 6-month-old *Spp1*^*fl/fl*^ and cKO mice (*n* = 3). **b** Quantification of a cytokine array in CEP tissues from 6-month-old *Spp1*^*fl/fl*^ and cKO mice (*n* = 4). **c** TRAP staining of bone marrow-derived macrophages (BMDMs) that had been treated with conditioned medium from the shNC or shOPN EPCs. **d** Tube formation assay in HUVECs that had been treated with conditioned medium from shOPN or shNC EPCs (*n* = 3). **e** Migration assay in BMDMs that had been treated with conditioned medium from the shOPN or shNC EPCs (*n* = 3). **f** ELISA quantification of IL-1β, IL-6, TNF-α, RANKL, VEGF, CCL2, and CCL5 levels in conditioned medium from the shOPN or shNC EPCs (*n* = 3). **g** WB analyses of OPN, CCL2, and CCL5 expression in the shOPN or shNC EPCs (*n* = 3). **h**, **i** BMDM migration assay using conditioned medium from the shOPN or shNC EPCs in the presence or absence of PF-4136309 (PF), maraviroc (MVC), or cenicriviroc (CVC) (*n* = 3). **j** IHC staining and quantification of CCL2 and CCL5 expression in CEP tissues from 6-month-old *Spp1*^*fl/fl*^ and cKO mice (*n* = 5). **k** WB analyses of CCL2 and CCL5 expression in CEP tissues from patients with mild or severe degeneration (*n* = 3). **l** IHC staining and quantification of CCL2 and CCL5 expression in CEP tissues from patients with mild or severe degeneration (*n* = 5). *P < 0.05, **P < 0.01, ***P < 0.001, ns: not significant

Based on the above results, it is reasonable to speculate that conditional *Spp1* knockout leads to a degenerative niche in the CEP. In support of this point, RT-qPCR analyses of CEP tissues from 6-month-old *Spp1*^*fl/fl*^ and cKO mice exhibited that the relative levels of gene transcripts, such as inflammation-related *Il1b, Il6, Il8, Tnf*, osteoclast activity-associated *Tnfsf11, Csf1, Tnfsf11b*, angiogenesis factors of *Vegfa, Pdgfb*, and *Ccl2, Ccl5, Cxcl1, Cxcl10* chemokines, were markedly higher in the CEPs from the cKO mice than those from *Spp1*^*fl/fl*^ mice (Fig. 4a). A similar pattern of IL-1β, IL-6, CXCL10, CXCL1, CCL2, CXCL2, CCL5, and TNF-α protein expression was detected by cytokine array between the cKO and *Spp1*^*fl/fl*^ mice, indicating that OPN deficiency promoted the formation of an inflammatory microenvironment in the CEP (Fig. 4b). However, the conditioned medium form OPN-silenced EPCs (shOPN) failed to enhance the osteoclastogenesis of bone marrow-derived macrophages (BMDMs) and the tube formation of HUVECs compared with that from the control cells (shNC) (Fig. 4c, d and Fig. S3B, C), but significantly promoted the migration of BMDMs (Fig. 4e). We then measured several cytokines involved in these processes using enzyme-linked immunosorbent assay (ELISA) and WB (Fig. 4f, g and Fig. S3D, E). The results indicated that OPN silencing did not significantly alter the levels of IL-1β, IL-6, TNF-α, RANKL, and VEGF in the supernatants of cultured EPCs, but increased the levels of CCL2 and CCL5, two important chemokines for the migration of macrophages.^36^ Moreover, treatment with PF-4136309 (PF, a selective CCR2 antagonist), maraviroc (MVC, a selective CCR5 antagonist), or cenicriviroc (CVC, a dual CCR2/CCR5 antagonist) obviously mitigated the macrophage migration induced by conditioned medium of cultured OPN-silenced EPCs (Fig. 4h, i and Fig. S3F, G). Similarly, the conditioned medium from primary EPCs isolated from cKO mice could also induce macrophage migration through CCL2 and CCL5 while displaying no significant effect on osteoclastogenesis or angiogenesis (Fig. S3H–K). Notably, the levels of CCL2 and CCL5 expression in the CEP of cKO mice were significantly higher than that in the *Spp1*^*fl/fl*^ mice, and they were significantly higher in human degenerative CEP tissues from the severe group than that in those from the mild group (Fig. 4j–l). Together, these data indicate that OPN deficiency enhances CCL2 and CCL5 expression in EPCs to recruit macrophages, disrupting CEP homeostasis.

### Macrophages perform distinct functions in a spatiotemporal pattern during the process of CEP remodeling

**Fig. 5:**
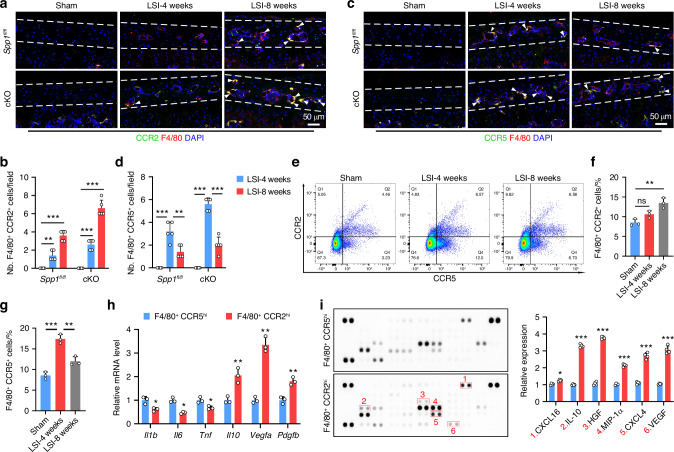
Macrophages perform distinct functions in a spatiotemporal pattern during the process of CEP remodeling. **a**, **b** Co-immunostaining images and quantification of F4/80^+^ (red) CCR2^+^ (green) macrophages in CEP tissues from the *Spp1*^*fl/fl*^ and cKO mice at 4 and 8 weeks post sham or LSI surgery (*n* = 5). **c**, **d** Co-immunostaining images and quantification of F4/80^+^ (red) CCR5^+^ (green) macrophages in CEP tissues from the *Spp1*^*fl/fl*^ and cKO mice at 4 and 8 weeks post sham or LSI surgery (*n* = 5). Cell sorting (**e**) and quantification of F4/80^+^CCR2^+^ (**f**) and F4/80^+^CCR5^+^ (**g**) macrophages in CEP tissues from cKO mice at 4 and 8 weeks post sham or LSI surgery (*n* = 3). **h** Relative levels of *Il1b, Il6, Tnf*, *Il10, Vegfa*, and *Pdgfb* mRNA transcripts in F4/80^+^CCR2^hi^ and F4/80^+^CCR5^hi^ macrophages (*n* = 3). **i** Quantification of an angiogenesis array in cell lysates from F4/80^+^CCR2^hi^ and F4/80^+^CCR5^hi^ macrophages (*n* = 4). *P < 0.05, **P < 0.01, ***P < 0.001, ns: not significant

It is possible that macrophages may serve as the predominant effectors for CEP remodeling. Hence, we examined the spatiotemporal changes of macrophages in the CEP during degeneration induced by LSI surgery. Consistent with previous observations, the number of F4/80^+^ macrophages significantly increased at 4- and 8-weeks post LSI surgery in both *Spp1*^*fl/fl*^ and cKO mice (Fig. 5a–d). Moreover, co-immunostaining analysis revealed a time-dependent increase in the number of F4/80^+^CCR2^+^ macrophages (Fig. 5a, b). Interestingly, the number of F4/80^+^CCR5^+^ macrophages increased at 4 weeks but declined at 8 weeks post the LSI (Fig. 5c, d). In addition, these changes were more evident in the CEP of cKO mice than *Spp1*^*fl/fl*^ mice and were further validated by flow cytometry analysis of F4/80^+^ macrophages in OPN-deficient CEP (Fig. 5e–g). Next, we tested the function of these two subtypes of macrophages. We purified these two subtypes of macrophages by fluorescence-activated cell sorting and analyzed them by RT-qPCR and a protein array. As expected, compared with F4/80^+^CCR5^hi^ macrophages, lower levels of IL-1β, IL-6, and TNF-α, but higher levels of IL-10, VEGF, and HGF were detected in F4/80^+^CCR2^hi^ macrophages (Fig. 5h, i), suggesting that F4/80^+^CCR5^hi^ macrophages may be predominant inflammatory factors for establishing an inflammatory niche at the beginning of CEP degeneration, while F4/80^+^CCR2^hi^ macrophages may mainly participate in angiogenesis at the latter stage. Thus, different types of macrophages perform distinct functions in a spatiotemporal pattern during the process of CEP remodeling.

### Macrophages activate the NLRP3 inflammasome in OPN-deficient EPCs

**Fig. 6:**
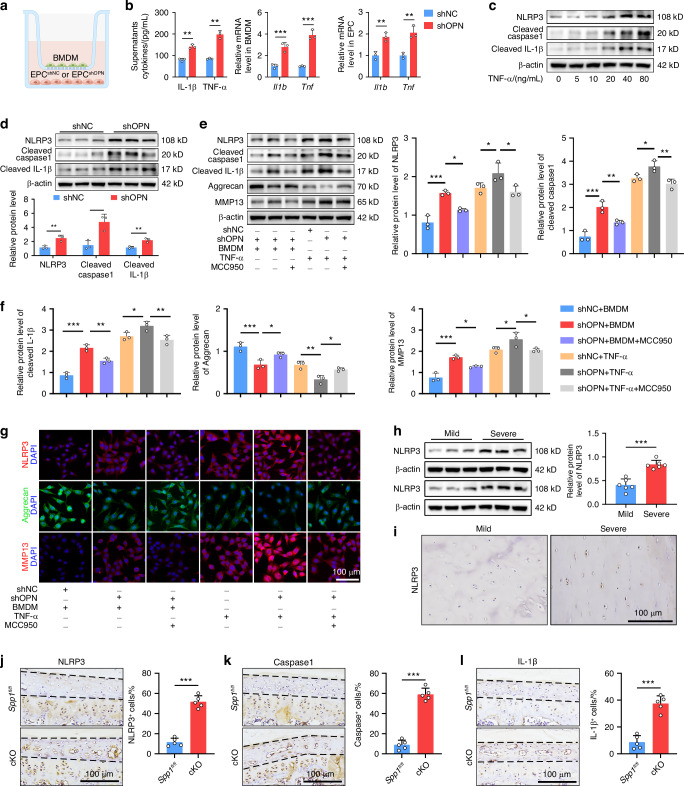
Macrophages activate the NLRP3 inflammasome in OPN-deficient EPCs. **a** A schematic graph of the co-culture system in which BMDMs were co-cultured with shOPN or shNC EPCs. **b** ELISA quantification of the levels of IL-1β and TNF-α in the supernatants of co-cultured cells (left); the relative levels of *Il1b* and *Tnf* mRNA transcripts in BMDMs (middle) and EPCs (right) in the coculture system (*n* = 3). **c** WB analyses of NLRP3, cleaved Caspase1, and cleaved IL-1β expression in EPCs in the absence or presence of different doses of TNF-α. **d** WB analyses of NLRP3, cleaved Caspase1, and cleaved IL-1β expression in shOPN or shNC EPCs after cocultured with BMDMs (*n* = 3). **e**, **f** WB analyses of NLRP3, cleaved Caspase1, cleaved IL-1β, Aggrecan, and MMP13 expression in shOPN or shNC EPCs that had been pretreated with or without MCC950 and then cocultured with BMDMs or treated with TNF-α (*n* = 3). **g** Representative IF staining of NLRP3, Aggrecan, and MMP13 in shOPN or shNC EPCs that had been pretreated with or without MCC950 and then cocultured with BMDMs or treated with TNF-α. **h**, **i** WB and IHC analyses of NLRP3 expression in CEP tissues from patients with mild or severe degeneration (*n* = 6). **j**–**l** IHC staining and quantification of NLRP3, Caspase1, and IL-1β expression in CEP tissues from 6-month-old *Spp1*^*fl/fl*^ and cKO mice (*n* = 5). *P < 0.05, **P < 0.01, ***P < 0.001

Previous scRNA-seq studies have suggested that intercellular communication between macrophages and IVD cells may contribute to IDD progression.^37,38^ Accordingly, we established a coculture system by co-culture of BMDMs with shOPN EPCs or shNC EPCs (Fig. 6a). ELISA revealed that the levels of IL-1β and TNF-α, the two most significant cytokines in IDD,^3^ in the shOPN group were significantly higher than those in the shNC group (Fig. 6b). A similar pattern of IL-1β and TNF-α mRNA transcripts was detected in both BMDMs and EPCs by RT-qPCR (Fig. 6b). These results implied that OPN silencing in EPCs modulated the co-cultured macrophages toward pro-inflammatory phenotype, and their interaction contributed to an inflammatory microenvironment. It is recognized that activation of the NLRP3 inflammasome promotes IVD inflammation and degeneration.^39^ Next, we tested whether OPN silencing modulated the activation of NLRP3 inflammasome in EPCs. While treatment with TNF-α increased the relative levels of NLRP3, cleaved Caspase1, and cleaved IL-1β expression in EPCs in a dose-dependent manner (Fig. 6c), these NLRP3 inflammasome-related proteins were clearly higher in the shOPN group than those in the shNC group (Fig. 6d), highlighting that the NLRP3 inflammasome was activated in shOPN EPCs after cocultured with BMDMs. Moreover, treatment with MCC950, a selective inhibitor of NLRP3, significantly mitigated the NLRP3 inflammasome activation and MMP13 expression in shOPN EPCs after cocultured with BMDMs or treated with TNF-α (Fig. 6e, f). In contrast, MCC950 treatment significantly rescued the expression of Aggrecan in both the co-cultured EPCs and the TNF-α-treated OPN-silenced EPCs (Fig. 6e, f). The protective effects of MCC950 on NLRP3 inflammasome activation, Aggrecan, and MMP13 expression in EPCs were observed by IF staining (Fig. 6g). Notably, the relative levels of NLRP3 expression in CEP tissues from the severe group were significantly higher than those in the mild group of patients (Fig. 6h, i). In addition, IHC unveiled that the number of NLRP3^+^, Caspase1^+^, and IL-1β^+^ EPCs in cKO mice was greater than that in *Spp1*^*fl/fl*^ mice (Fig. 6j–l). Collectively, the data indicate that OPN deficiency enhances the activation of NLRP3 inflammasome in EPCs following interaction with macrophages.

### OPN deficiency promotes macrophage migration and NLRP3 activation in the CEP by enhancing the NF-κB signaling

**Fig. 7:**
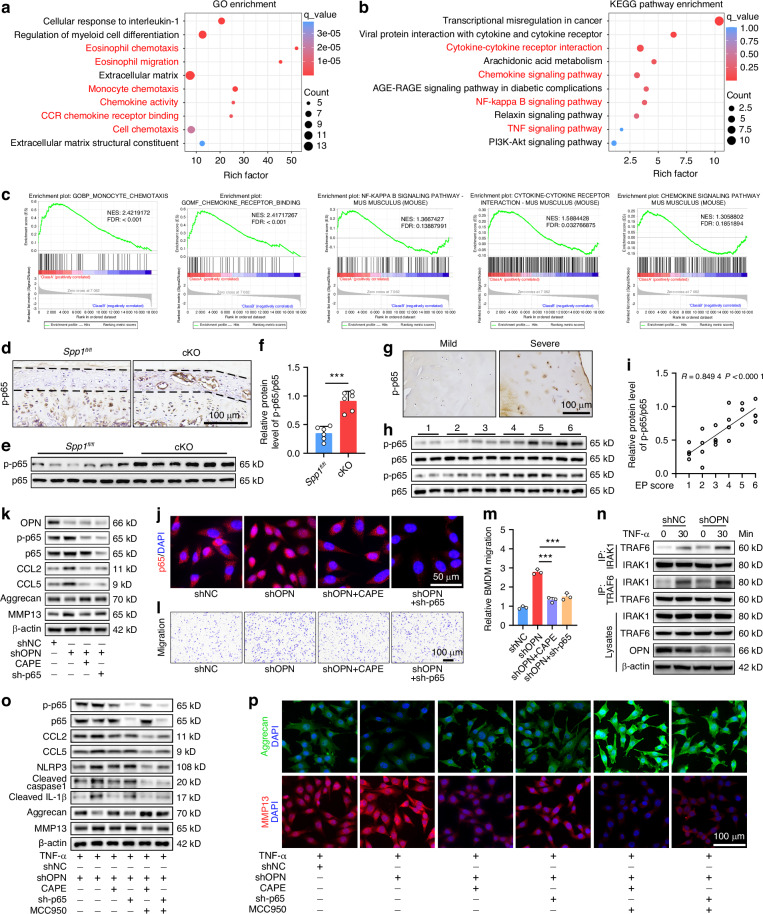
OPN deficiency promotes macrophage migration and NLRP3 activation in the CEP by enhancing the NF-κB signaling. The GO (**a**) and KEGG (**b**) enrichment analysis of the differentially expressed genes (DEGs) in CEP tissues from 6-month-old *Spp1*^*fl/fl*^ and cKO mice. **c** The gene set enrichment analysis of the DEGs. **d** IHC staining of phosphorylated p65 (p-p65) in CEP tissues from 6-month-old *Spp1*^*fl/fl*^ and cKO mice. **e**, **f** WB analysis of p-p65 levels in CEP tissues from 6-month-old *Spp1*^*fl/fl*^ and cKO mice (*n* = 5). **g** IHC staining of p-p65 in CEP tissues from patients with mild or severe degeneration. **h**, **i** WB analysis of p-p65 levels in human CEP tissues and their correlation with the severity of degeneration (*n* = 24). **j**–**k** IF staining and WB analyses of p65 in shOPN or shNC EPCs that had been pretreated with, or without, CAPE and transduced with, or without, lentivirus for p65-specific shRNA (sh-p65). **l**, **m** BMDM migration toward the conditioned medium from shNC or shOPN EPCs that had been transduced with, or without, lentivirus for the expression of sh-p65 and then pretreated with, or without, CAPE (*n* = 3). **n** Co-IP analysis of the effect of OPN deficiency on IRAK1-TRAF6 interaction in EPCs. **o** WB analyses of p-p65, p65, CCL2, CCL5, NLRP3, cleaved Caspase1, cleaved IL-1β, Aggrecan, and MMP13 expression in shOPN or shNC EPCs that had been transduced with, or without, lentivirus for the expression of sh-p65, pretreated with, or without, CAPE and MCC950, and then treated with TNF-α. **p** IF staining of Aggrecan and MMP13 in shOPN or shNC EPCs that had been transduced with, or without, lentivirus for the expression of sh-p65, pretreated with, or without, CAPE and MCC950, and then treated with TNF-α. ***P < 0.001

To identify the molecular mechanisms by which OPN deficiency influences CEP homeostasis, the transcriptome profiles in CEP tissues from 6-month-old *Spp1*^*fl/fl*^ and cKO mice were analyzed and there were remarkably differentially expressed genes (DEGs) between them (Fig. S4A, B). The Gene Ontology (GO) analysis of DEGs indicated that OPN deficiency enhanced cell chemotaxis (Fig. 7a). Meanwhile, the Kyoto Encyclopedia of Genes and Genomes (KEGG) revealed that the DEGs were enriched in the inflammation pathways, including the TNF, nuclear factor kappa B (NF-κB), and chemokine signaling pathways, as well as cytokine-cytokine receptor interaction (Fig. 7b). Moreover, the gene set enrichment analysis (GSEA) substantiated that OPN deficiency favored the formation of an inflammatory niche in the CEP (Fig. 7c). Accordingly, the NF-κB pathway might be a crucial mechanism underlying the action of OPN deficiency in CEP degeneration of cKO mice. Actually, WB and IHC assays unveiled that the relative levels of NF-κB p65 phosphorylation (p-p65) were significantly higher in the cKO mice than those in the *Spp1*^*fl/fl*^ mice (Fig. 7d–f), as well as in human CEP tissues from the severe group than those in the mild group (Fig. 7g, h). Remarkably, the relative levels of p-p65 in CEP samples were positively correlated with EP scores in this population of patients (Fig. 7i). In parallel, WB and IF assays revealed that OPN silencing enhanced the NF-κB signaling and MMP13 expression, but reduced Aggrecan expression in EPCs, which were mitigated or abrogated by treatment with caffeic acid phenethyl ester (CAPE, an inhibitor of NF-κB) or by NF-κB p65 silencing (Fig. 7j, k). In addition, treatment with CAPE or NF-κB p65 silencing in shOPN EPCs dramatically decreased the CCL2 and CCL5 expression, and mitigated the BMDM migration induced by conditioned medium from cultured OPN-silenced EPCs (Fig. 7k–m). Clearly, OPN deficiency enhanced the NF-κB signaling and increased CCL2 and CCL5 expression in EPCs to recruit macrophages, contributing to inflammation in the CEP.

Given that aberrant mechanical stress is a significant contributor to CEP degeneration and IDD progression,^40^ we next evaluate the roles of OPN in a mechanical stress model induced by intermittent cyclic mechanical tension (ICMT), which is a common way to mimic the in vivo mechanical strain experienced by EPCs.^41^ The results showed that both TNF-α and ICMT treatments could significantly decrease the expression level of OPN while increasing the protein levels of NLRP3 and p-p65, and these trends were more obvious following co-treatment with TNF-α and ICMT (Fig. S4E). Moreover, the addition of recombinant OPN protein could reverse the TNF-α- and ICMT-induced upregulation of NLRP3 and p-p65, respectively, reducing the production of IL-1β but enhancing the expression of SOX9 (Fig. S4F, G). These findings further confirmed the critical role of OPN in maintaining CEP homeostasis under inflammatory stimulation or abnormal mechanical stress, especially in regulating the NF-κB/NLRP3 axis.

It is reported that IRAK1 acts as a transcription factor of the *Il10* gene in the presence of OPN to control hyper-inflammation,^42^ and the formation of the IRAK1-TRAF6 complex is one of the key steps for activating the NF-κB signaling.^43^ Conceivably, whether OPN could modulate the interaction between endogenous IRAK1 and TRAF6 in EPCs was examined by co-immunoprecipitation (Co-IP) assays. It was noted that OPN exhibited no interaction with IRAK1 or TRAF6 under normal conditions but coimmunoprecipitated with IRAK1 in the presence of TNF-α (Fig. S4H), which was also supported by the immunofluorescent colocalization of OPN and IRAK1 in TNF-α-treated EPCs (Fig. S4I). While TNF-α stimulation induced the formation of the IRAK1-TRAF6 complex in shNC EPCs, the amount of precipitated TRAF6 by anti-IRAK1 or precipitated IRAK1 by anti-TRAF6 in the shOPN EPCs was significantly more than that in the shNC cells (Fig. 7n), suggesting that OPN might compete with IRAK1 or TRAF6 for their interactions to disrupt their complex assembly. More importantly, treatment with CAPE or NF-κB p65 silencing as well as together MCC950 abrogated the TNF-α-induced p65 phosphorylation and NLRP3 inflammasome activation, mitigating IL-1β and Caspase1 cleavage as well as CCL2, CCL5, and MMP13 expression, but enhancing Aggrecan expression in the OPN-silenced EPCs (Fig. 7o, p and Fig. S4C). Together, these data unveil that OPN deficiency activates the NF-κB signaling by enhancing the interaction between IRAK1 and TRAF6 to increase CCL2/CCL5 production, thereby promoting macrophage migration and the NLRP3 activation, and disrupting ECM homeostasis in the CEP.

### Pharmacological inhibition of the NF-κB/NLRP3 axis attenuates CEP degeneration in cKO mice following LSI

**Fig. 8:**
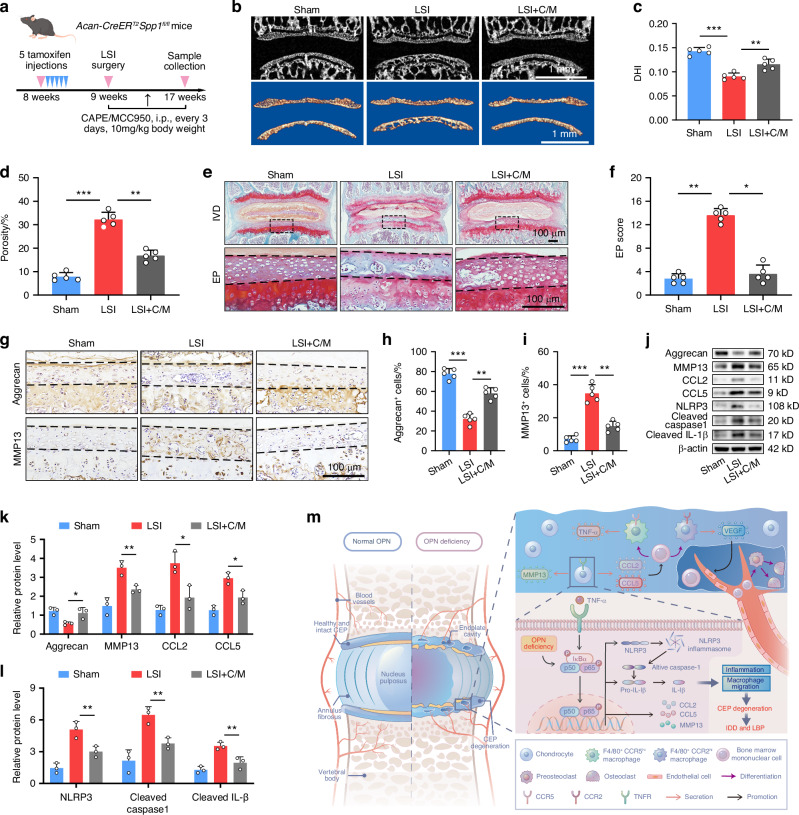
Pharmacological inhibition on the NF-κB/NLRP3 axis attenuates CEP degeneration in cKO mice following LSI. **a** A schematic graph illustrates the experimental design. The cKO mice at 8 weeks of age were treated with tamoxifen for 5 consecutive days to induce *Spp1* knockout and at 9 weeks of age, the mice were randomized and subjected to a sham or LSI surgery. Subsequently, the LSI cKO mice were treated intraperitoneally with vehicle or MCC950 and CAPE (C/M) every three days for 8 weeks. **b** μCT (top) and reconstruction (bottom) images of the coronal plane of lumbar endplates in cKO mice. Quantification of μCT analyses for DHI (**c**) and the porosity (**d**) of mouse lumbar endplates in cKO mice (*n* = 5). SO-FG staining (**e**) and histological scores (**f**) of lumbar EP in cKO mice (*n* = 5). **g**–**i** IHC staining and quantification of Aggrecan and MMP13 expression in CEP tissues from cKO mice (*n* = 5). **j**–**l** WB analyses of Aggrecan, MMP13, CCL2, CCL5, NLRP3, cleaved Caspase1, and cleaved IL-1β expression in CEP tissues from cKO mice (*n* = 3). **m** A schematic illustration of the mechanism by which OPN deficiency influences CEP homeostasis by enhancing the NF-κB signaling during the process of IDD. *P < 0.05, **P < 0.01, ***P < 0.001

Our findings indicate that OPN deficiency promotes CEP degeneration by enhancing the NF-κB signaling to recruit macrophages and activate the NLRP3 inflammasome. Finally, the effects of treatment with both CAPE and MCC950 (C/M) on the LSI-induced CEP degeneration in cKO mice were tested (Fig. 8a). The μCT analysis exhibited that treatment with C/M partially mitigated the pathological changes induced by LSI in the CEP, improving the DHI and reducing the porosity of endplates (Fig. 8b–d). In addition, Safranine O-Fast Green staining revealed that the C/M treatment remarkably ameliorated the progression of endplate sclerosis and decreased the EP histological scores in LSI-treated cKO mice (Fig. 8e, f). Mechanistically, the C/M treatment alleviated LSI-induced ECM catabolism and IVD inflammation by increasing Aggrecan, decreasing MMP13, CCL2, CCL5, NLRP3, cleaved Caspase1, and cleaved IL-1β expression in the CEP of cKO mice following LSI (Fig. 8g–l). Therefore, these data indicate that pharmacological inhibition on the NF-κB signaling and NLRP3 inflammasome activation by the C/M treatment effectively attenuates inflammation and CEP degeneration in cKO mice following LSI.

## Discussion

Although the CEP is viewed as an unremarkable structure in the IVD, recent advances have demonstrated that the CEP is critical for maintaining IVD homeostasis and its dysfunction contributes to the pathogenesis of IDD and LBP.^20,34,44–46^ Therefore, understanding the role of the CEP in physiological disc homeostasis and pathogenesis of IDD may help in uncovering novel targets as well as therapeutic strategies for the clinical treatment of IDD. With the scRNA-seq technology, several studies have constructed comprehensive transcriptomic landscapes of IVD tissue at a single-cell level, revealing the cellular heterogeneity and communication network within the IVD niche.^9–12^ Notably, a recent conjoint analysis has uncovered that the essential pathogenic factors in different cell subpopulations influence CEP homeostasis, among which OPN is critical.^31^ Additionally, another scRNA-seq analysis of cells from IDD patients has proposed that OPN level could serve as a new biomarker for the evaluation of IDD,^30^ suggesting a tight connection between OPN and IDD. Thus, OPN may affect IDD progression by regulating CEP homeostasis, and understanding the mechanisms underlying the action of OPN in the progression of IDD may help in developing innovative approaches for IDD therapy.

OPN is a non-collagenous and multifunctional glycoprotein expressed in various tissues and cell types. Functionally, OPN can influence bone metabolism and homeostasis by regulating biological processes of BMSCs, hematopoietic stem cells, chondrocytes, osteoblasts, and osteoclasts.^24,47^ Importantly, OPN is recognized as a significant regulator of the development and progression of many bone diseases.^24^ Actually, the roles of OPN in the pathogenesis of bone diseases are controversial. While OPN retarded osteoarthritis progression through the OPN/CD44/PI3K signaling,^26^ inhibiting the PI3K/AKT pathway could suppress cartilage degeneration and subchondral bone remodeling in osteoarthritis.^25^ Similarly, two research groups have reached opposite conclusions regarding the role of OPN in IDD. Consistent with our findings, Tomaszewski et al. observed that OPN expression was down-regulated in degenerated and calcified endplates,^27^ while Xiao et al. found that OPN expression was up-regulated in ossified endplates of IDD.^28^ It is possible that OPN from different cell populations may have various biological effects during the process of different stages of degeneration. Therefore, we should investigate the function of OPN in specific tissues and cell types.

This study showed that OPN was mainly decreased in the CEP during degeneration and its expression levels were negatively correlated with the degree of CEP damage. Meanwhile, we found a slight decrease in OPN expression in NP, which is far less obvious than the findings by Zhou et al.^29^ From our point of view, this is due to the different animal models that were used. In their study, the significant decrease in OPN expression in NP could be attributed to the direct damage to NP caused by the needle puncture. Additionally, they suggested that OPN might suppress hypertrophy and vascularization while stimulating chondrogenesis in NP, indicating a potential role of OPN in NP homeostasis as well as different roles in different IVD cells, which requires more detailed investigations in future studies. Still, given the remarkable difference in OPN expression in CEP, we employed the LSI model in this work and intended to study the role of OPN in the CEP niche using conditional OPN knockout mice. Notably, we observed spontaneous degenerative changes with age in the CEP of cKO mice, including ECM degradation, cell death, and chondrocyte hypertrophy, which preceded the degeneration of NP/AF and the whole IVD, highlighting the importance of CEP integrity for IVD homeostasis.

CEP is believed to play a crucial role in the IVD by equalizing the load between the IVD and vertebral body and preserving a physiological hydrostatic pressure within the NP to prevent water loss under mechanical stress.^48^ However, CEP disappears progressively with aging and degeneration, which can be attributed to the apoptosis of EPCs, according to Ariga et al.^49^ They found that the apoptosis of EPCs increased with aging and worsened in the surgery-induced degenerative CEP, aligning with our results. Notably, the widespread presence of apoptosis in the CEP was not contemporaneously seen in the NP,^49^ suggesting that the pathological alterations in the CEP may begin first and lead to subsequent NP degeneration. Indeed, endplate damage in IDD patients has been reported to cause necrosis and apoptosis in the neighboring NP,^50^ whereas endplate injury in animal models triggers IDD similar to that seen in humans.^51^ Specifically, endplate defects can contribute to a significant decrease in the amount of proteoglycan while increasing the expression levels of matrix degrading enzymes, pro-inflammatory factors, and pro-apoptotic proteins in the IVD, leading to degenerative changes.^52,53^ Furthermore, such defects facilitate the intrusion of NP tissue into the vertebral body, resulting in a lower pressure in NP but a higher stress on the surrounding AF.^54,55^ It is noteworthy that a single impact injury without structural damages on the endplate is sufficient to initiate IDD,^56^ which is commonly seen in the clinic.^57^ Additionally, due to the accumulation of mechanical loading, microstructural damage occurs before any major alterations in native collagen composition, which is thought to be the cause of the earliest changes in the mechanical properties of CEP.^58^ Subsequently, the changed mechanical strain negatively impacts the metabolism of IVD cells and may cause the development of “endplate-driven IDD”.^59,60^ More importantly, the population-based studies have demonstrated that the endplate defect is strongly linked to IDD across all lumbar disc levels, suggesting it as an initiation factor in IDD.^60–62^ Hence, based on the above findings as well as ours, it is fair to conclude that CEP degeneration could promote the occurrence and development of IDD.

In this study, we observed significant degeneration of CEP in the abnormal mechanical stress-induced mouse IDD model through LSI surgery, which was further exacerbated by the conditional deletion of OPN in the IVD. Notably, both the hypertrophic and the apoptotic chondrocytes decreased, rather than increased, in cKO mice, relative to the *Spp1*^*fl/fl*^ mice following LSI. According to the results reported by Yang et al. and Zhou et al.^63,64^ hypertrophic chondrocytes can undergo transdifferentiation into osteoblasts and osteocytes in addition to apoptosis in endochondral bone formation. To some extent, the CEP degeneration is similar to endochondral bone formation. Hence, the reduced number of hypertrophic and apoptotic chondrocytes may be attributed to the completion of endochondral bone formation in cKO mice at 8 weeks post LSI surgery. We are interested in further investigating the dynamic process of the cell fate of EPCs following CEP degeneration using the lineage tracing method in a transgenic mouse model.

OPN deficiency in the IVD of adult mice can induce endochondral ossification in the CEP, along with the ingrowth of type H blood vessels, high osteoclast activity, and macrophage infiltration, implying the occurrence of CEP remodeling. Previous studies have revealed that excessive loading and biomechanical instability can cause local tissue injury and altered microenvironment, promoting an osteoclast-initiated remodeling process and the eventual development of IDD.^6,65,66^ In parallel, we found that OPN deficiency exacerbated the degenerative remodeling process in the CEP of mice following LSI. Particularly, OPN deficiency enhanced the bone resorption-mediated by osteoclasts in the CEP of cKO mice following LSI, evidenced by the increased number of porosity and osteoclasts. However, these findings were different from previous studies which suggested that OPN knockout compromised bone resorption in mice subjected to ovariectomy.^67–69^ The different observations may stem from the different stains of mice. While our study employed *Spp1* knockout in Aggrecan-expressing cells, their study used systemic *Spp1* knockout mice. It is well known that OPN derived from different types of cells has diverse biological functions. For example, OPN in chondrocytes can accelerate their proliferation,^70^ but M1 macrophage-derived OPN promotes chondrocyte apoptosis during the process of osteoarthritis.^71^ In addition, macrophage-secreted OPN can induce excessive angiogenesis and activate osteoclasts to foment bone matrix degradation in high-fat diet-fed mice.^47^ The osteoclasts’ initial attachment to the bone surface and their subsequent absorption activity depend on the OPN-related intracellular signaling.^72^ Moreover, up-regulated OPN expression enhances the proliferation and migration of MSCs at resorption sites on the bone surface during remodeling.^73^ Collectively, these findings suggest that OPN may contribute significantly to CEP remodeling. However, further investigations are necessary for understanding how OPN expression is regulated in different types of cells, such as MSCs, osteoblasts, and macrophages.

Endothelial cells, osteoclasts, and BMDMs are involved in the process of CEP remodeling and disc degeneration in both animal models and IDD patients.^34,35,66,74–76^ Similarly, we observed positive staining of CD31, TRAP, and F4/80 in human CEP specimens (Fig. S5A–C), laying a solid foundation for the conclusion of this study. Specifically, the degenerated disc is capable of generating substantial quantities of proinflammatory, pro-osteoclastic chemokines, and cytokines, which can readily diffuse from the disc into the neighboring bone marrow,^76–78^ causing chemotaxis gradients and immune cell activation. Consequently, the enhanced osteoclastic activity contributes to a porous endplate that allows for the ingrowth of blood vessels,^34,66^ and the activated immune cells exacerbate IDD even without infiltrating the disc.^79^ Strikingly, the conditioned medium from OPN-deficient EPCs could not induce angiogenesis or osteoclastogenesis in vitro but could attract the migration of macrophages through high levels of CCL2 and CCL5, suggesting that macrophages might serve as downstream predominant effectors. Macrophages are resident in many organs and tissues to support homeostasis, development, and regeneration.^80^ Interestingly, we found that M1-like F4/80^+^CCR5^hi^ macrophages and M2-like F4/80^+^CCR2^hi^ macrophages participated in early phase and later phase of CEP remodeling in mice in a spatiotemporal pattern. Consistently, these different subtypes of macrophages regulate bone homeostasis and periosteal bone formation with a similar functional pattern.^47,80^ However, given the complex immune landscape in IDD,^37,38^ we are interested in further investigating the influence of other subtypes of macrophages and immune cells on CEP homeostasis in future studies.

The healthy and intact IVD is regarded as an immune privilege organ due to its unique structure.^81^ However, following the disruption of IVD homeostasis, immune cells can infiltrate into IVD tissues, altering the microenvironment and biofunctions of IVD cells.^37,38^ In this work, we observed higher levels of IL-1β and TNF-α expression in CEP tissues from cKO mice and in OPN-deficient EPCs that were co-cultured with macrophages, suggesting that the cellular interaction contributes significantly to the formation of an inflammatory niche. It is recognized that the NLRP3 inflammasome activation is crucial for IL-1β production and IVD inflammation.^39^ Similar to the findings in NP degeneration,^82^ high activation of the NLRP3 inflammasome was detected in EPCs both in vitro and in vivo, which was effectively suppressed by MCC950. Further experiments revealed that the NLRP3 activation was closely related to the NF-κB signaling, of which the inhibition on the NF-κB signaling protected EPCs against TNF-α-induced injury. Moreover, treatment with both CAPE and MCC950 protected against the TNF-α-induced inflammation in OPN-deficient EPCs and attenuated CEP degeneration in cKO mice following LSI, implying that the NLRP3 inflammasome might also be regulated by other pathways, besides the NF-κB, including the mTOR and FOXO3a signaling and others.^83,84^ Notably, OPN can inhibit the NLRP3 inflammasome activity in other disease models.^85,86^ Therefore, clarifying the relationship between OPN and NLRP3 may provide new insights into the OPN regulation on CEP homeostasis, thereby developing biomedical treatments for IDD.

Previous studies demonstrated that there was a positive inflammatory feedback loop in the CEP, which promoted inflammation independently from the disc and the bone marrow,^87–89^ suggesting that progressive CEP damage can result from both mechanical factors and local inflammatory mediators within the bone marrow and CEP. In this work, we observed an obvious decline in OPN expression level as well as an inhibitory effect of recombinant OPN protein on the activation of the NF-κB/NLRP3 axis under inflammatory or mechanical stress, further validating our hypothesis that OPN deficiency facilitates the activation of the NF-κB/NLRP3 axis in degenerated EPCs. However, considering the difficulties in local drug delivery in the CEP of the mouse lumbar spine and the diversity of ways in which OPN functions, we did not evaluate the therapeutic effect of OPN-targeted therapy in IDD mice, such as local injection of OPN-expressing adeno-associated virus or recombinant OPN protein, which becomes a shortcoming of this study as well as our future research focus. Furthermore, we did not determine the initial events that down-regulate OPN expression. In addition to alternative splicing, OPN can undergo significant posttranslational modifications, such as sulfation, phosphorylation, and glycosylation, and modulation of these processes has the potential to influence OPN activity, which requires more investigations during the IDD process. Therefore, we insist that developing OPN-targeted therapy in IDD should be based on a comprehensive understanding of the biofunctions and underlying mechanisms of OPN, not only in CEP but also in other IVD tissues. Still, this study demonstrated that OPN deficiency promoted CEP degeneration by enhancing NF-κB signaling to recruit macrophages and activate the NLRP3 inflammasome (Fig. 8m), suggesting an avenue for OPN-based therapeutic strategies for IDD.

## Materials and methods

### Human samples

The CEP specimens were obtained from 24 patients (10 females and 14 males) with degenerative disc disease or scoliosis in the Orthopedics Department of Xinqiao Hospital, following the protocol approved by the Ethics Committee of Xinqiao Hospital (2022-YD212-01) and the guidelines of the Declaration of Helsinki. The informed consent was obtained from each donor before conducting this study. These patients were then assessed for their endplates on T1-weighted magnetic resonance imaging (MRI) scans and the damage severity of each endplate was scored according to a previous report.^33^ The detailed information of human samples was provided in Table S1. Briefly, the samples scoring 1 were only used in Western blot for correlation analysis, while other samples scoring over 1 were used in both Western blot and immunohistochemistry experiments, categorized as mild (scored 2 or 3) and severe (scored 4, 5, or 6) groups.

### Animals

C57BL/6 mice were obtained from Hunan SJA Laboratory Animal Limited Company (Hunan, China). The *Acan-CreER*^*T2*^ (stock no. 019148) mice were purchased from the Jackson Laboratory (Bar Harbor, USA). The *Spp1*^*fl/fl*^ mice (homozygous for the Spp1 fled allele) in C57BL/6 background were provided by Cyagen Biosciences (Suzhou, China) using standard procedures. The *Spp1*^*fl/fl*^ mice were crossed with *Acan-CreER*^*T2*^ mice to get *Acan-CreER*^*T2*^*Spp1*^*fl/+*^ mice, which were further backcrossed with *Spp1*^*fl/fl*^ mice to generate *Acan-CreER*^*T2*^*Spp1*^*fl/fl*^ mice. Their genotypes were determined by PCR analysis using the following primers: *Acan-CreER*^*T2*^: common: 5′-AAAAGCGACAAGAAGACACCA-3′, mutant forward: 5′-CTCCAGACTGCCTTGGGAAAA-3′, wild type forward: 5′-GTTATATTCCGGAGCCCACA-3′; *Spp1*^*fl/fl*^: forward: 5′-TAGTGCCACATGTGACTTTAGTCTT-3′, reverse:5′-TTCTGGATTTATCTCAGCCCCTC-3′. To achieve conditional deletion of *Spp1* in Aggrecan-expressing cells, 8-week-old *Acan-CreER*^*T2*^*Spp1*^*fl/fl*^ male mice were intraperitoneally administered with tamoxifen (MedChemExpress, HY-13757A, 1 mg/10 g body weight) daily for 5 consecutive days. The age-matched *Spp1*^*fl/fl*^ male mice treated with tamoxifen were used as control mice. All mice on a C57BL/6 background were housed in a specific pathogen-free facility of Army Medical University. To establish a mouse model of IDD induced by lumbar spine instability (LSI), nine-week-old *Acan-CreER*^*T2*^ *Spp1*^*fl/fl*^ male mice were anesthetized intraperitoneally with tribromoethanol (Sigma-Aldrich, T48402, 4 mg/10 g body weight), and resected their lumbar^*3rd*^-lumbar^*5th*^ (L3-L5) spinous processes along with the supraspinous and interspinous ligaments. The control mice received a sham surgery with only detaching the posterior paravertebral muscles from the L3-L5 vertebrae. The experimental mice were randomized and injected intraperitoneally with 10 mg/kg body weight MCC950 (Selleck, S7809) and CAPE (Selleck, S7414) or an equivalent volume of phosphate-buffered saline (PBS) every 3 days for 8 weeks. All in vivo experiments were approved by the Laboratory Animal Welfare and Ethics Committee of Army Medical University (AMUWEC20227510).

### Isolation and culture of mouse endplate chondrocytes (EPCs)

EPCs were isolated from the CEP of each 5-day-old male C57BL/6 mouse. Briefly, CEP tissues were dissected and cut into small pieces using ophthalmological scissors. The tissues were digested with 0.2% type II collagenase (Gibco, 17101015) in DMEM/F12 (Gibco, 11330032) at 37 °C for 30 min at a shaking speed of 210 r/min. The cell suspension was filtered through a 70 μm cell strainer (Corning, 352350) into a clean tube, and the remaining tissues were washed twice with precooled cell-suspension buffer (5% fetal bovine serum [FBS; Gibco, 10100147 C] in PBS). The buffer was also collected in the same tube, which was centrifuged at 500 *g* for 5 min. The cell pellet was resuspended in 2 mL of cell-suspension buffer and stored on ice. The remaining tissues were repeatedly digested again to improve the yield of primary EPCs. The generated EPCs were cultured in complete DMEM/F12 medium containing 10% FBS and 1% penicillin/streptomycin (Beyotime, C0222) at 37 °C with 5% CO_2_. EPCs were exposed to the fresh medium every 3 days and passaged at 80% confluence using 0.25% trypsin (HyClone, SH30042.01). The first three passages of EPCs cultured in a monolayer were used in this study. To induce an abnormal mechanical stress, EPCs were cultured on a six-well BioFlex plate and subjected to an intermittent cyclic mechanical tension (ICMT) at 0.5 Hz with 10% elongation for 12 h/day for 2 days using the FX-5000T Flexcell Tension Plus system (Flexcell International Corporation, USA).

### Western blot (WB)

CEP tissues and EPCs were homogenized and lysed in the lysis buffer (Beyotime, P0013) on ice for 30 min, respectively. The lysate samples (20–30 μg/lane) were electrophoresed on 4%–20% SurePage Gels (GenScript, M00656) and transferred onto Polyvinylidene Fluoride membranes (Merck-Millipore, MA, USA). The membranes were blocked with Blocking Buffer (Beyotime, P0252) and then incubated with primary antibodies (1:1 000 dilution) overnight at 4 °C. The antibodies against OPN (Proteintech, 22952-1-AP), β-actin (Beyotime, AF0003), Aggrecan (Proteintech, 13880-1-AP), NF-κB p65 (Proteintech, 10745-1-AP), SOX9 (Millipore, AB5535), CCL2 (Proteintech, 25542-1-AP), CCL5 (Affinity Biosciences, AF5151), NLRP3 (Affinity Biosciences, DF7438), Collagen II (ABclonal, A1560), Caspase1 (ABclonal, A0964), MMP13 (Proteintech, 18165-1-AP), IL-1β (CST, 12242), and Phospho-NF-κB p65 (CST, 3033) were used. After being washed, the membranes were incubated with corresponding secondary antibodies. Then the bands were detected using SuperSignal West Pico PLUS Chemiluminescent Substrate (Thermo, 34580) and monitored on the Vilber chemiluminescence imaging system (FX6. Edge, France). Finally, the ratios of each protein band intensity to the control β-actin were analyzed using ImageJ software (NIH, MD, USA).

### Co-immunoprecipitation (Co-IP)

The direct interaction between proteins was determined by Co-IP using a Dynabeads™ Protein G Immunoprecipitation Kit (Thermo, 10007D), according to the manufacturer’s protocol. Briefly, individual protein lysates (1 mg each) were reacted with 5 µg anti-IRAK1 (Santa Cruz, sc-5288) or anti-TRAF6 (Santa Cruz, sc-8409) antibodies that had been coated on 1.5 mg Dynabeads at 4 °C overnight. After washed with cold IP lysis buffer, the immunocomplex on the beads were eluted by boiling in Laemmli sample buffer and subjected to WB.

### Enzyme-linked immunosorbent assay (ELISA)

The levels of cytokines in the supernatants were analyzed using commercial kits for IL-1β (Proteintech, KE10003), IL-6 (Proteintech, KE10007), TNF-α (Proteintech, KE10002), RANKL (Proteintech, KE10035), VEGF (Proteintech, KE10009), CCL2 (Proteintech, KE10006) and CCL5 (Proteintech, KE10017) according to the manufacturers’ instructions.

### Histochemistry, immunohistochemistry (IHC), and immunofluorescence (IF)

Human CEP tissues and mouse lumbar spines were fixed in 4% PFA (Biosharp, BL539A) for 24–48 h and decalcified in 10% EDTA (Mengbio, MBB013). The samples were embedded in paraffin or OCT compound (Sakura Finetek, 4583) and cut in coronal sections. The paraffin tissue sections (4 μm) of the L4-L5 lumbar spine were routinely stained with Safranine O-Fast Green (Solarbio, G1371), TRAP (Servicebio, G1050), and subjected to immunohistochemistry using a standard methodology. The middle part of the lower CEP was selected as the region of interest in order to maintain the consistency of the experimental results. The pathological changes in individual tissue sections were histologically scored. Frozen sections (30 μm) were standardly stained with fluorescent antibodies for analysis of blood vessels, while additional frozen sections (10 μm) were prepared for other IF staining. The sections were incubated with primary antibodies against Collagen X (1:100, Abcam, ab58632), Aggrecan (1:100, Proteintech, 13880-1-AP), Collagen II (1:100, Santa Cruz, sc-52658), OPN (1:100, Proteintech, 22952-1-AP), MMP13 (1:100, Proteintech, 18165-1-AP), F4/80 (1:50, Invitrogen, 14-4801-82), CD31 (1:50, R&D Systems, AF3628), Endomucin (1:50, Santa Cruz, sc-65495), CCL2 (1:100, Proteintech, 25542-1-AP), CCL5 (1:100, Affinity Biosciences, AF5151), CCR5 (1:100, ABclonal, A20261), CCR2 (1:100, Abcam, ab216863), NLRP3 (1:100, Affinity Biosciences, DF7438), Caspase1 (1:100, ABclonal, A0964), IL-1β (1:100, CST, 12242), and Phospho-NF-κB p65 (1:100, Santa Cruz, sc-136548). After being washed, the samples were incubated with corresponding secondary antibodies for 1 h at 37 °C. The IHC sections for immunoactivity were visualized with a DAB Substrate Kit (ZSGB-BIO, ZLI-9018), and counterstained with hematoxylin (Solarbio, G1080). The sections for IF staining were mounted with Antifade Mounting Medium (Beyotime, P0131) before being captured under an inverted microscope (Nikon Ts2-FL, Japan), and a fluorescence microscope (Zeiss Axio Observer 7, Germany), respectively. The images were quantified using ImageJ software.

### Real time quantitative polymerase chain reaction (RT-qPCR)

Total RNA was extracted with a kit (BioFlux, BSC52S1). After quantitation by a NanoDrop One spectrophotometer (Thermo, MA, USA), 1 μg of each RNA sample was reverse-transcribed into complementary DNA with the RevertAid Master Mix (Thermo, M16325). The relative levels of interesting gene mRNA transcripts to the control GAPDH were determined by RT-qPCR using the SYBR Green qPCR SuperMix (Invitrogen, 11744500) on a LightCycler 96 RT-PCR system (Roche, Switzerland). Data were calculated by the 2^−ΔΔCt^ method.

### Micro-computed tomography (μCT) analysis

Mice were euthanized and their whole lumbar spine tissues were dissected, followed by fixing in 4% PFA for 24–48 h. The tissue samples in PBS were examined using a high-resolution μCT scanner (Bruker, SkyScan1272) with a voltage of 65 kV and a current of 153 μA. Images at a resolution of 5.0 μm per pixel were reconstructed using NRecon v1.7 and analyzed by CTAn v1.2. Five consecutive images were used to display a three-dimensional reconstruction of the vertebrae and endplates with the visualization software CTvox v3.3. Data included following parameters: disc height, disc height index (DHI), trabecular number (Tb. N), trabecular thickness (Tb. Th), bone volume fraction (BV/TV), cortical thickness, and porosity.^34,90^

### Cytokine and angiogenesis arrays

The levels of 40 different chemokines and cytokines in tissue lysates of endplates were analyzed by a cytokine array (R&D, ARY006). The levels of angiogenic factors in cell lysates of macrophages were characterized using a Mouse Angiogenesis Array Kit (R&D, ARY015). The immunoreactive dots were detected and quantified using the Vilber chemiluminescence imaging system (FX6. Edge, France).

### In vitro assays for BMDM migration, osteoclast differentiation, and tube formation

Bone marrow cells were flushed from the hindlimbs of male C57BL/6 mice (6–10 weeks old) with DMEM/F12. The cell suspensions were filtered through a 70 μm cell strainer and centrifuged at 300 g for 5 min. After lysis of red blood cells with ACK lysis buffer (Beyotime, C3702), the remaining cells were resuspended in complete DMEM/F12. After culture for 4 h, the non-adherent cells were collected and cultured in complete DMEM/F12 containing 30 ng/mL M-CSF (SinoBiological, 51112-MNAH) for 7 days to obtain BMDMs. In the migration assay, 2 × 10^5^ BMDMs in FBS-free DMEM/F12 medium were cultured in the upper chamber of a 6-well Transwell plate (Corning, 3428), and the conditioned medium containing test reagents was added to the bottom chamber. After culturing for 24 h, the migrated BMDMs on the bottom surface of upper chamber membranes were fixed in 4% PFA for 15 min and permeabilized with absolute methanol for 20 min at room temperature. After removing the BMDMs on the upper surface of the upper chamber membranes, the migrated BMDMs were stained with crystal violet (Beyotime, C0121) for 10 min and photoimaged under a microscope. For osteoclast differentiation, 2 × 10^5^ BMDMs were cultured in 6-well plates in the conditioned medium for 4 days. The cells were fixed in 4% PFA and stained for TRAP activity using a specific reagent, according to the manufacturer’s instructions. Osteoclasts were identified to have >3 nuclei positive for TRAP staining and counted in blinded manner. In the tube formation assay, HUVECs (2 × 10^4^ cells/well) were cultured in the conditioned medium for 6 h in precooled 96-well plates that had been coated with 50 μL of dissolved Matrigel (Corning, 356231) at 37 °C for 30 min. The tube formation in each well was evaluated and imaged under an inverted microscope (Nikon Ts2-FL, Japan), followed by quantifying with ImageJ software.

### Flow cytometry and cell sorting

The CEP tissues were dissected at the indicated time points post LSI surgery and used for preparation of single-cell suspension. The CEP cells were stained with APC-anti-mouse F4/80 (Biolegend, 123116), PE/Cyanine5-anti-mouse CD11b (Biolegend, 101209), PE-anti-mouse CCR5 (Biolegend, 107005), and FITC-anti-mouse CCR2 (Biolegend, 150607) for 20 min in the dark on ice, followed by staining with DAPI (Biolegend, 422801). All of the above antibodies were diluted at a ratio of 1:200. The samples were stained with the same isotype antibodies and served as the negative controls. The cells were gated on live cells and specific types of cells were sorted in a FACSAria III cell sorter (BD Biosciences, CA, USA) or analyzed using BD FACSDiva software (BD Bioscience) and FlowJo software (BD Bioscience).

### Lentivirus transduction

The lentiviral-based shRNAs for OPN (shOPN), p65 (sh-p65), and a negative control (shNC) were designed and synthesized by Tsingke Biotechnology Co. (Beijing, China) and were cloned into pLKO.1-TRC vectors using the target sequences provided in Supplementary Table 2. EPCs were cultured in 6-well plates until 50% ~ 70% confluence and transduced with the lentivirus at a MOI of 10 for 12 h, following the manufacturer’s instructions. At 48 h after transfection, EPCs expressing the shRNA plasmids were selected by 2 μg/mL puromycin and further subjected to WB analysis for determining transfection efficiency (Figs. S3A, S4D).

### RNA sequencing and analysis

RNA sequencing was performed by Sinotech Genomics (Shanghai, China). Firstly, total RNA was extracted from CEP tissues using Trizol, and after quality and quantification of RNA samples in an Agilent 2100 Bioanalyzer (Agilent Technologies, CA, USA) using a Qubit^®^ 3.0 Fluorometer (Life Technologies, CA, USA) and a Nanodrop One spectrophotometer (Thermo, MA, USA), the RNA samples were reverse-transcribed to generate cDNA libraries. After purification, the libraries were quantified by a Qubit^®^ 3.0 Fluorometer and validated in an Agilent 2100 Bioanalyzer, followed by sequencing in an Illumina NovaSeq 6000 (Illumina, USA). A multiple-hypothesis test was used to correct the p-value, and the threshold of the p-value was determined by controlling the false discovery rate (FDR). The differentially expressed genes (DEGs) with a FDR < 0.05 and the absolute value of log_2_ fold change (log_2_FC) ≥ 1 were analyzed using the edgeR package. Finally, DEGs were analyzed by the Kyoto Encyclopedia of Genes and Genomes (KEGG), Gene Ontology (GO), and GSEA.

### Statistical analysis

All data are reported as mean ± standard deviation. The difference between groups was analyzed by unpaired two-tailed Student’s *t*-test and the data among multiple groups were analyzed by the one-way or two-way ANOVA and post hoc Tukey’s test using GraphPad Prism 9.0 (GraphPad Software). *P* values of less than 0.05 were considered statistically significant.

## Supplementary information

Supplementary material

## Data Availability

All data are available from the corresponding authors upon reasonable request.
