# Supplementary material for: Osteopontin deficiency promotes cartilaginous endplate degeneration by enhancing the NF-κB signaling to recruit macrophages and activate the NLRP3 inflammasome
Source: Bone Res. 2024 Sep 6;12:53. doi: 10.1038/s41413-024-00355-3 (PMC11379908; doi:10.1038/s41413-024-00355-3)
Supplement: Supplementary file 1 — Supplementary material [file 41413_2024_355_MOESM1_ESM.docx]

**Supplementary material**

**
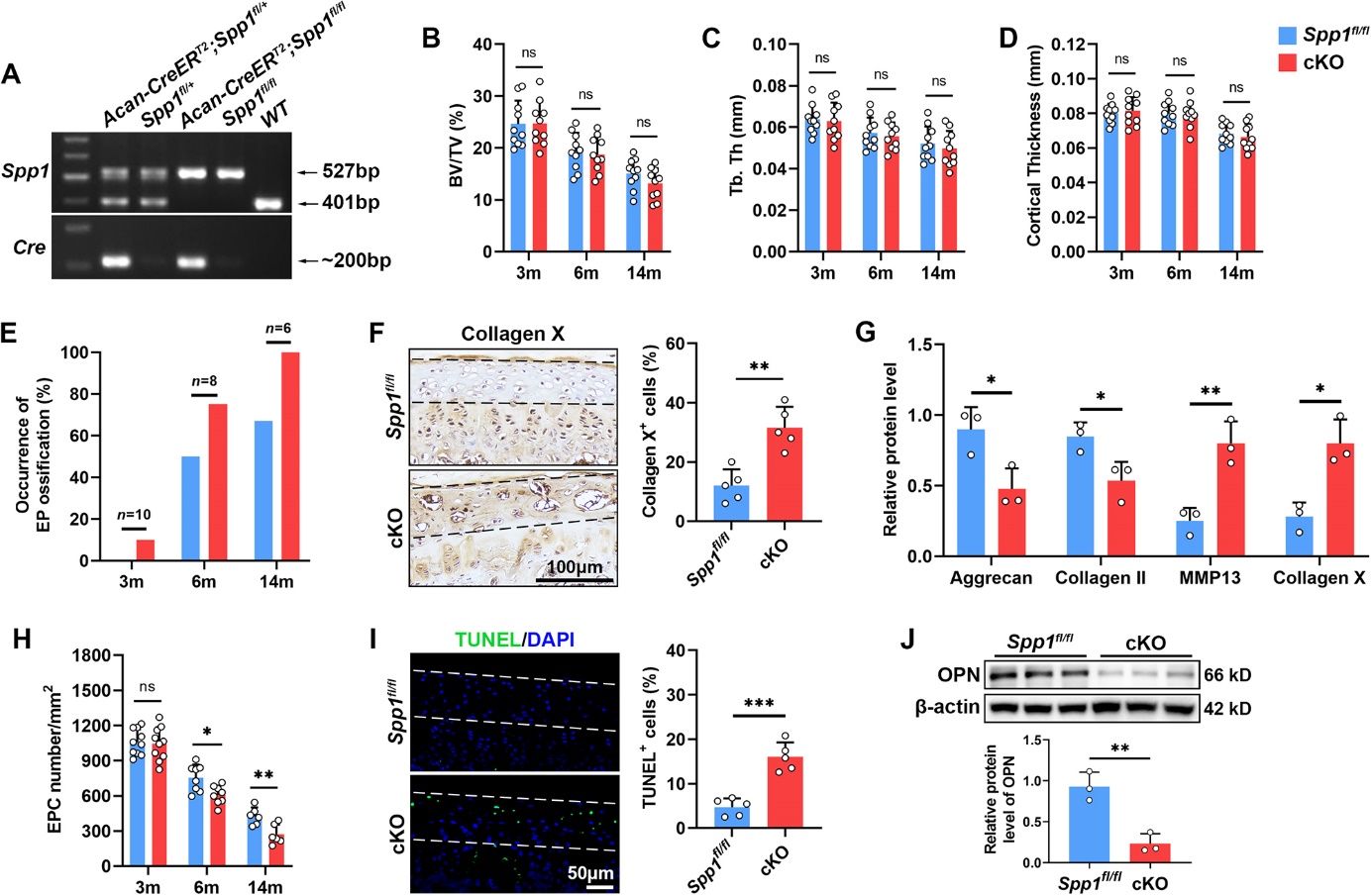
**

**Figure S1. OPN deficiency deteriorates the age-related CEP degeneration. (A)** Genotype identification by PCR. **(B–D)** Quantification of μCT analyses for bone volume/trabecular volume (BV/TV; **B**), trabecular thickness (Tb. Th; **C**), and cortical thickness (**D**) of the lumbar vertebrae from 3, 6, and 14-month-old *Spp1^fl/fl^* and cKO mice. *n* = 10. **(E)** Occurrence of CEP ossification in 3, 6, and 14-month-old *Spp1^fl/fl^* and cKO mice. **(F)** IHC staining and quantification of Collagen X expression in CEP tissues from 6-month-old *Spp1^fl/fl^* and cKO mice. *n* = 5. **(G)** Quantification of WB analyses of Aggrecan, Collagen II, MMP13, and Collagen X expression in CEP tissues from 6-month-old *Spp1^fl/fl^* and cKO mice. *n* = 3. **(H)** The number of EPCs in CEP tissues from 3 (*n* = 10), 6 (*n* = 8), and 14-month-old (*n* = 6) *Spp1^fl/fl^* and cKO mice. **(I)** TUNEL analysis of apoptotic cells in CEP tissues from 6-month-old *Spp1^fl/fl^* and cKO mice. *n* = 5. **(J)** WB analysis of OPN expression in CEP tissues from 3-month-old *Spp1^fl/fl^* and cKO mice. *n* = 3. **P <* 0.05, ***P <* 0.01, ****P <* 0.001, ns: not significant.


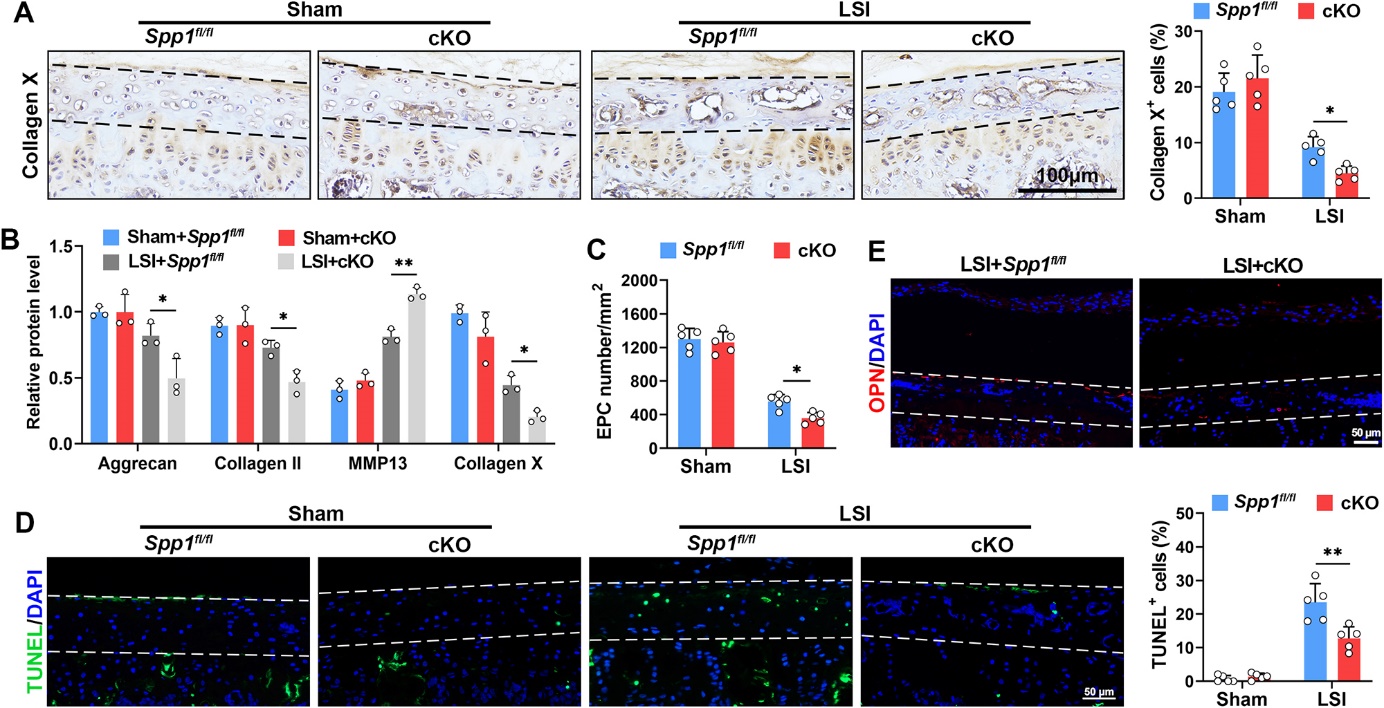


**Figure S2. OPN deficiency accelerates CEP remodeling in mice following LSI.** **(A)** IHC analysis of Collagen X expression in CEP tissues from *Spp1^fl/fl^* and cKO mice with sham or LSI surgery. *n* = 5. **(B)** Quantification of WB analyses of Aggrecan, Collagen II, MMP13, and Collagen X expression in CEP tissues from *Spp1^fl/fl^* and cKO mice with sham or LSI surgery. *n* = 3. **(C)** The number of EPCs in CEP tissues from *Spp1^fl/fl^* and cKO mice with sham or LSI surgery. *n* = 5. **(D)** TUNEL analysis of apoptotic cells in CEP tissues from *Spp1^fl/fl^* and cKO mice with sham or LSI surgery. **(E)** IF staining of OPN expression in the IVD tissues from *Spp1^fl/fl^* and cKO mice following LSI. **P <* 0.05, ***P <* 0.01.


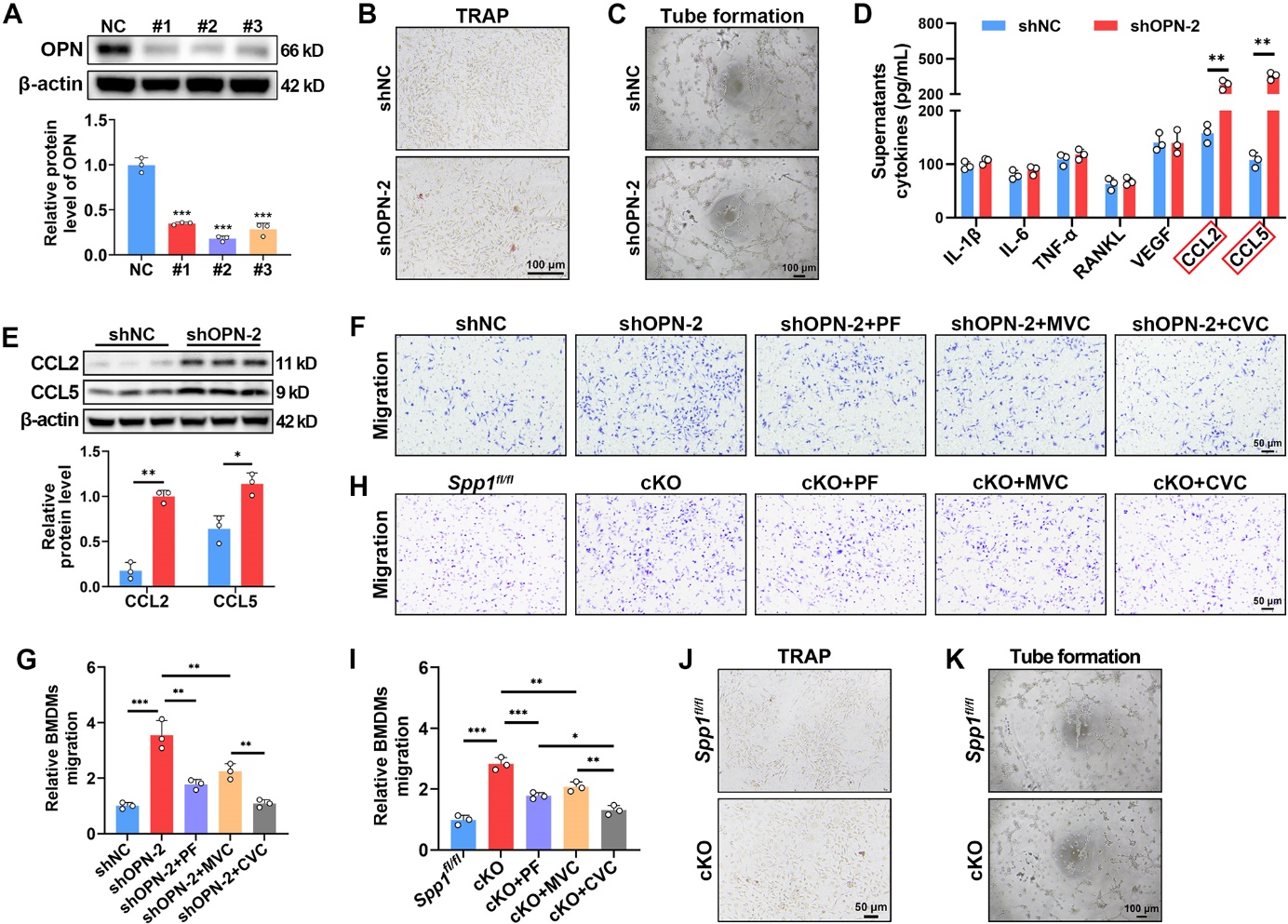


**Figure S3. OPN-deficient EPCs enhances CCL2 and CCL5 expression to recruit macrophages.** **(A)** WB analysis of transfection efficiency of lentiviral-based shRNA for OPN. *n* = 3. **(B-C)** TRAP staining and tube formation assay after treatment with conditioned medium from the shNC or shOPN-2 EPCs. **(D)** ELISA quantification of IL-1β, IL-6, TNF-α, RANKL, VEGF, CCL2, and CCL5 levels in conditioned medium from the shNC or shOPN-2 EPCs. *n* = 3. **(E)** WB analyses of CCL2 and CCL5 expression in the shNC or shOPN-2 EPCs. *n* = 3. **(F-G)** BMDM migration assay using conditioned medium from the shNC or shOPN-2 EPCs in the presence or absence of PF-4136309 (PF), maraviroc (MVC), or cenicriviroc (CVC). *n* = 3. **(H-I)** BMDM migration assay using conditioned medium from primary EPCs isolated from *Spp1^fl/fl^* and cKO mice in the presence or absence of PF, MVC, or CVC. *n* = 3. **(J-K)** TRAP staining and tube formation assay after treatment with conditioned medium from primary EPCs isolated from *Spp1^fl/fl^* and cKO mice. **P <* 0.05, ***P <* 0.01, ****P <* 0.001.


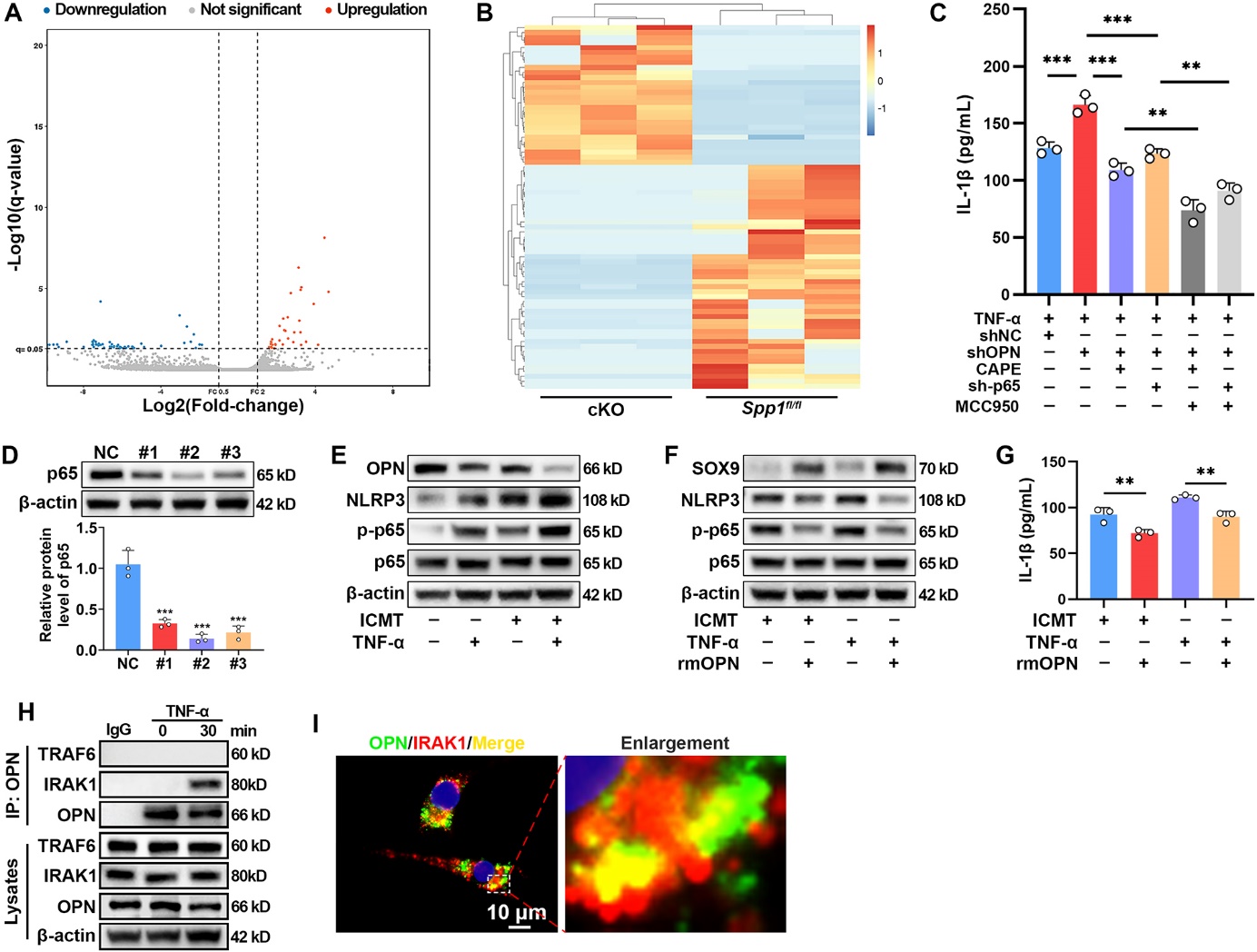


**Figure S4. OPN deficiency promotes macrophage migration and NLRP3 activation in the CEP by enhancing the NF-κB signaling.** **(A)** The volcano diagram of differentially expressed genes (DEGs) in CEP tissues from cKO and *Spp1^fl/fl^* mice. **(B)** Heatmap analysis of DEGs in CEP tissues from cKO and *Spp1^fl/fl^* mice. **(C)** ELISA quantification of IL-1β levels in culture medium from shOPN or shNC EPCs that had been transduced with, or without, lentivirus for the expression of sh-p65, pretreated with, or without, CAPE and MCC950, and then treated with TNF-α. *n* = 3. **(D)** WB analysis of transfection efficiency of lentiviral-based shRNA for p65. *n* = 3. **(E)** WB analyses of OPN, NLRP3, p-p65, and p65 expression in EPCs treated with TNF-α and/or intermittent cyclic mechanical tension (ICMT). **(F)** WB analyses of SOX9, NLRP3, p-p65, and p65 expression in EPCs pretreated with or without recombinant OPN protein, and then treated with TNF-α or ICMT. **(G)** ELISA quantification of IL-1β levels in culture medium from EPCs pretreated with or without recombinant OPN protein, and then treated with TNF-α or ICMT. *n* = 3. **(H)** Co-IP analysis of the interaction between OPN and IRAK1 or TRAF6 in response to TNF-α stimulation in EPCs. **(I)** Immunofluorescent colocalization of OPN and IRAK1 in TNF-α-treated EPCs. ***P <* 0.01, ****P <* 0.001.


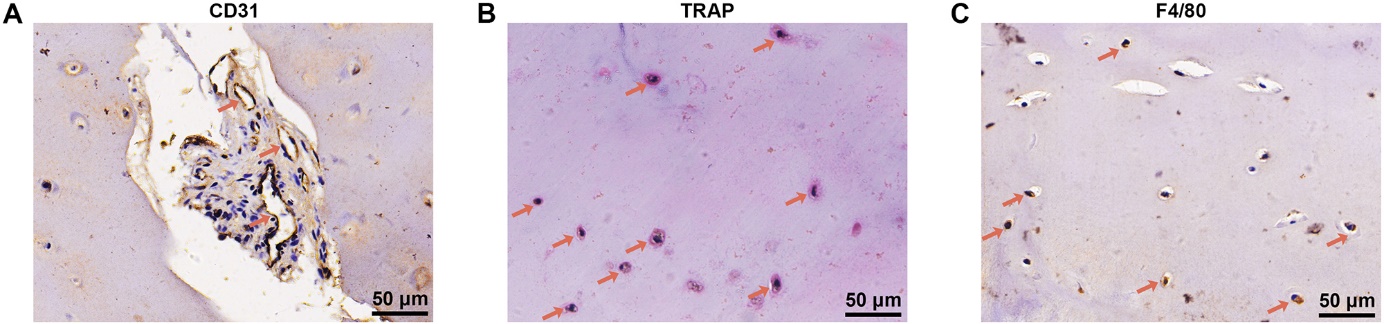


**Figure S5. (A)** IHC staining of CD31 in human CEP specimen. **(B)** TRAP staining in human CEP specimen. **(C)** IHC staining of F4/80 in human CEP specimen.

| **Donor** | **Age** | **Gender** | **Diagnosis** | **Surgical segment** | **Endplate score** | **Application** |
| --- | --- | --- | --- | --- | --- | --- |
| Donor 1 | 30 | Male | LDH | L5-S1 | 3 | WB/IHC |
| Donor 2 | 27 | Male | LDH | L4-5 | 2 | WB |
| Donor 3 | 27 | Male | LDH | L5-S1 | 2 | WB/IHC |
| Donor 4 | 55 | Female | LS | L5-S1 | 4 | WB |
| Donor 5 | 31 | Male | LDH | L5-S1 | 3 | WB |
| Donor 6 | 39 | Female | LDH | L4-5 | 2 | WB/IHC |
| Donor 7 | 29 | Male | LDH | L4-5 | 1 | WB |
| Donor 8 | 81 | Male | LDH | L5-S1 | 6 | WB/IHC |
| Donor 9 | 78 | Female | LSS | L5-S1 | 5 | WB |
| Donor 10 | 84 | Male | LS | L4-5 | 5 | WB/IHC |
| Donor 11 | 70 | Male | LDH | L3-4 | 4 | WB |
| Donor 12 | 65 | Female | LSS | L4-5 | 5 | WB |
| Donor 13 | 22 | Female | LS | L5-S1 | 3 | WB/IHC |
| Donor 14 | 79 | Female | LSS | L3-4 | 6 | WB |
| Donor 15 | 82 | Male | LSS | L4-5 | 4 | WB/IHC |
| Donor 16 | 25 | Male | LDH | L4-5 | 2 | WB |
| Donor 17 | 79 | Female | LDH | L4-5 | 5 | WB/IHC |
| Donor 18 | 26 | Male | LSS | L5-S1 | 1 | WB |
| Donor 19 | 74 | Male | LS | L3-4 | 6 | WB |
| Donor 20 | 28 | Male | LDH | L5-S1 | 1 | WB |
| Donor 21 | 68 | Male | LSS | L4-5 | 4 | WB/IHC |
| Donor 22 | 81 | Female | LS | L5-S1 | 6 | WB |
| Donor 23 | 76 | Female | LSS | L4-5 | 3 | WB/IHC |
| Donor 24 | 30 | Female | LDH | L4-5 | 1 | WB |
| LDH, lumbar disc herniation; LSS, lumbar spinal stenosis; LS, lumbar spondylolisthesis; WB, western blot; IHC, immunohistochemistry | | | | | | |

**Supplementary Table 1. Detailed information of human samples used in the study**

| **shRNA** | **Target sequence** |
| --- | --- |
| shOPN-1 | CCGGCCGAGGTGATAGTGTGGTTTACTCGAGTAAACCACACTATCACCTCGGTTTTTT |
| shOPN-2 | CCGGAGTTGAATGGTGCATACAACTCGAGTTGTATGCACCATTCAACTTTTTTT |
| shOPN-3 | CCGGCGAGGAGTTGAATGGTGCATACTCGAGTATGCACCATTCAACTCCTCGTTTTTT |
| sh-p65-1 | GCCTTAATAGTAGGGTAAGTT |
| sh-p65-2 | CGGATTGAGGAGAAACGTAAA |
| sh-p65-3 | GCAGGCTATCAGTCAGCGCAT |

**Supplementary Table 2. shRNA target sequences**
